# Emotion prediction as computation over a generative theory of mind

**DOI:** 10.1098/rsta.2022.0047

**Published:** 2023-07-24

**Authors:** Sean Dae Houlihan, Max Kleiman-Weiner, Luke B. Hewitt, Joshua B. Tenenbaum, Rebecca Saxe

**Affiliations:** ^1^ Department of Brain and Cognitive Sciences, Massachusetts Institute of Technology, Cambridge, MA, USA; ^2^ Department of Psychology, Harvard University, Cambridge, MA, USA

**Keywords:** emotion, inverse planning, theory of mind, social intelligence, affective computing, probabilistic generative model

## Abstract

From sparse descriptions of events, observers can make systematic and nuanced predictions of what emotions the people involved will experience. We propose a formal model of emotion prediction in the context of a public high-stakes social dilemma. This model uses inverse planning to infer a person’s beliefs and preferences, including social preferences for equity and for maintaining a good reputation. The model then combines these inferred mental contents with the event to compute ‘appraisals’: whether the situation conformed to the expectations and fulfilled the preferences. We learn functions mapping computed appraisals to emotion labels, allowing the model to match human observers’ quantitative predictions of 20 emotions, including joy, relief, guilt and envy. Model comparison indicates that inferred monetary preferences are not sufficient to explain observers’ emotion predictions; inferred social preferences are factored into predictions for nearly every emotion. Human observers and the model both use minimal individualizing information to adjust predictions of how different people will respond to the same event. Thus, our framework integrates inverse planning, event appraisals and emotion concepts in a single computational model to reverse-engineer people’s intuitive theory of emotions.

This article is part of a discussion meeting issue ‘Cognitive artificial intelligence’.

## 1. Introduction

Human social life depends on our ability to understand and anticipate other people’s emotions. Intense efforts in both basic science and industrial applications are currently directed towards building models of emotion recognition: identifying a person’s emotions from their expression. Here we tackle a complementary challenge: predicting how a person will emotionally react to an event.

To illustrate the phenomenon we target, imagine watching an episode of the popular British gameshow called ‘Golden Balls’ [1]. During the episode, two players, Arthur and Bella, play a public one-shot social game called ‘Split or Steal’. On the table is a pot of $100 000 USD. Eventually, each player will secretly choose to Split (Cooperate) or Steal (Defect). If both players choose to Split, each takes home $50k. If both choose to Steal, they both leave with nothing. But if one chooses to Split and the other chooses to Steal, the one who stole takes the entire $100k and the other player leaves with nothing.^1^ Before Arthur and Belle make their choices, the gameshow host gives them a chance to talk to each other (in front of the live studio audience and TV viewers at home). They both vehemently promise to choose Split. Then they each make their secret choice. The choices are revealed simultaneously—they both chose Split! What do you predict Arthur will feel in this moment? Even without seeing their expressions, human observers generate systematic predictions about others’ emotional reactions to events [3–5]. For example, observers predict Arthur will feel *joy*, *relief* and *gratitude*. By contrast, if he Split but Bella Stole, Arthur is predicted to feel *disappointment*, *envy* and *contempt*.

The question for the current research is: How do human observers generate these emotion predictions? Social games offer a highly constrained but emotionally evocative context for studying social cognition. The ‘Split or Steal’ game can be fully described by a simple set of variables but evokes diverse and fine-grained predictions of players’ emotions. We develop a Bayesian framework [6,7] to formalize the conceptual knowledge, and the cognitive reasoning, that observers use to predict others’ emotional reactions to events [8]. We aim to capture how observers generate abstract representations of others’ minds from situational cues, tailor their emotion predictions to a specific individual, and predict distinctively social emotions like *guilt*, *embarrassment* and *respect*.

**Figure 1.**
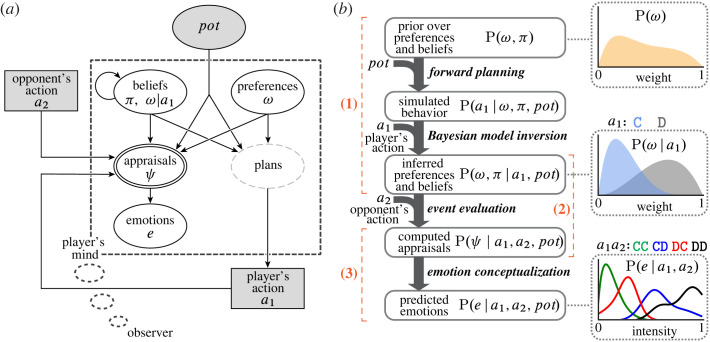
Emotion prediction as inference over an intuitive Theory of Mind. Hypotheses about how human observers reason about others’ emotions can be formalized as probabilistic generative models. This reflects a hypothesis about observers’ intuitive theory of other people’s minds, not a scientific hypothesis about people’s actual emotions. (*a*) Implementation of the general hypothesis for the ‘Split or Steal’ game (a public one-shot Prisoner’s Dilemma). We treat observers’ emotion predictions as a function of their intuitive reasoning about how players will subjectively evaluate, or ‘appraise’, the game’s outcome. Observers predict a player’s emotions by inferring what preferences and beliefs motivated the player’s decision to Cooperate or Defect, and reason about how those preferences and beliefs would cause the player to emotionally react to the outcome of the game. The intuitive theories we test take the form of directed acyclic graphs, where arrows indicate the causal relationship between variables. Shaded nodes are observable variables and open nodes are latent variables. Round nodes are continuous variables, rectangular nodes are discrete variables. Nodes with a single border are random variables. The double border indicates that appraisals are calculated deterministically. Plans are shown with a partial border because they are not explicitly represented in this model. (*b*) Computational model of the intuitive theory. The model comprises three modules. Module (1) infers a joint distribution over preferences and beliefs given a player’s action via inverse planning. Module (2) computes appraisals based on how a player would evaluate the outcome of the game with respect to the inferred preferences and beliefs. Module (3) generates emotion predictions by transforming the computed appraisals. The probability density plots illustrate how observers’ prior belief about a player’s preference P(ω) is updated based on the player’s action, and how the inferred preference $P(\omega |a_{1})$ is used to predict the player’s emotional reaction to the game’s outcome $P(e|a_{1},a_{2})$.

Our model is organized around a psychological premise: observers predict Arthur’s emotional reaction by reasoning about how he will evaluate the situation relative to his desires and beliefs [8–14]. Accordingly, the overall model of emotion prediction (figure 1) comprises three Modules, which simulate how observers (1) infer Arthur’s preferences and beliefs, (2) reason about how Arthur will evaluate (or ‘appraise’) events with respect to his mental contents (e.g. Was the event something he expected and wanted to happen?), and (3) predict Arthur’s emotions based on his likely appraisals. Module (1) uses inverse planning to model how observers reason over an intuitive Theory of Mind (wherein a player chooses actions that maximize a subjective utility function) to infer a player’s mental states [15–17]. Module (2) computes appraisals by reasoning about how a player will evaluate an event based on his inferred mental contents. Module (3) translates the computed appraisals into predictions of the player’s emotions using learned emotion concepts (i.e. functions that express emotions in terms of appraisals).

This work aims to computationally recapitulate how human observers predict others’ emotional reactions. To fit and test the model, we collect high-resolution behavioural data on the ‘Split or Steal’ game. Observers on Amazon mTurk made systematic predictions of 20 nuanced emotions and 20 individual players. Combining the modules, we quantitatively simulate how human observers predict players’ fine-grained emotional reactions to hypothetical events.

## 2. Relation to prior work

To predict what emotions Arthur will experience when he splits the pot with Belle, observers reason about what the situation means to Arthur. We build a model that generates emotion predictions from a description of the events (i.e. the same information given to observers). There are many approaches to building situation-computable models of emotion prediction [18], including behavioural economic decision models [19,20], rule-based emotion schemas [21,22], multi-agent computer simulations [12,23,24] and large language models [25,26]. These methodologically and philosophically diverse approaches share a view that emotion prediction depends on abstracting Theory of Mind representations from contextual information about a situation. However, these approaches vary widely in how the models abstract, represent and reason over Theory of Mind information.

Our work foregrounds this critical process, treating the prediction of emotional reactions to events as cognitive reasoning based on the inference of preferences and beliefs. We frame human emotion understanding as inference over an *intuitive theory* of other minds. An intuitive theory is a logically and causally structured mental model (a ‘lay’ ontology) [27–29], which is typically not explicit or fully introspectable [30]. This work studies an aspect of the intuitive theory of emotion: observers’ mental model of people’s emotional reactions to events. Note that the aim of this work is to build a formal scientific model of people’s intuitive theory of emotion, not to test whether the intuitive theory is accurate. That is, although people are able to sensitively infer and accurately predict others’ emotions in some contexts [31–33], people make systematic errors in other contexts [3,34,35]. Because we are interested in capturing and characterizing people’s intuitive theory of emotion, we do not here attempt to test the ground truth accuracy of either the observers’ or the model’s predictions, only their similarity to each other.

### (a) Inverse planning

How do human observers infer a specific person’s preferences and beliefs? One source of information is the person’s actions. People typically choose intentional actions that are likely to achieve their goals or maximize their rewards, given their values and beliefs (the principle of rational action). As a result, even a single sparse observation (e.g. observing one action) can lead observers to update estimates of the person’s mental states [36,37]. Observers’ intuitive reasoning about others’ actions can be framed as a theory-based Bayesian model [6,7], which formalizes how causal structure and prior beliefs constrain inference of latent mental contents [16]. In models of ‘inverse planning’, a forward planning model simulates how approximately rational agents, imbued with rich cognitive structure, perceive, plan and act in a dynamic world. Bayesian inversion of the forward model then supports inverse inference of what unobserved mental contents were likely to have caused the observed behaviour. Inverse planning models can closely match how observers use others’ behaviour to infer mental contents, such as beliefs, preferences, rewards, costs, habits and intelligence [15,17,38–41].

Inverse planning has been extensively applied in the domain of action understanding [42–44]. Adapting inverse planning to predict nuanced social emotions imposes demands on the latent representations and computations beyond what are typically required for action understanding. Predicting emotions like *envy*, *guilt*, *respect* and *gratitude* requires representing multifaceted preferences and recursive beliefs about rewards, costs, interpersonal relationships and reputation. Predicting emotions like *surprise*, *disappointment*, *regret* and *relief* requires computing prediction errors and counterfactuals.

While there are numerous approaches to modelling Theory of Mind [45,46], our work makes a principled commitment to inverse planning [8]. Module (1) instantiates an inverse planning model that infers rich abstract Theory of Mind representations. The forward model formalizes our hypotheses about observers’ conceptual knowledge of others’ intentional actions. For instance, observers know that people’s choices reflect values beyond their own monetary gain, such as equity and achieving a desirable reputation [36,47,48]. We hypothesize that observers infer these values to predict social emotions. To capture emotion predictions that depend on social values, we incorporate weighted utility terms like equity and reputation into the model. We empirically test these hypotheses by comparing observers’ attributions of players motivations against the model’s inference, and subsequently use the inferred weights to capture observers’ predictions of nuanced social emotions.

### (b) Inferring appraisals

Module (2) extends the inverse planning framework, using the inferred preferences and beliefs to simulate observers’ latent reasoning about how a player will evaluate a new world state (the outcome of the game). The idea that emotional experience is a consequence of how people evaluate events with respect to their desires and beliefs is a central principle of Appraisal Theory [49–51]. Appraisal Theories describe first-person emotional experience as an interaction between a person and an event, with respect to *appraisal variables* such as goal congruence (Did she get what she wanted?), agency (Who caused the event, and with what intention?), controllability (Could she influence the situation?) and probability (How likely was the outcome?) [52,53].

Many researchers have proposed that observers reason about others’ emotions using a mental model of appraisal-like processes [4,8,10,12,54–58]. An influential theory is that people engage in a process of ‘reverse appraisal’ to infer the desires and beliefs of others by observing their emotional reactions to events [11,59,60]. Reverse appraisal emphasizes inverse inference: that is, reasoning about the appraisals that caused someone’s emotions and expressions [13,59]. Empirical work supports the idea that people infer appraisals from expressions [61] and, based on the inferred appraisals, update their understanding of others’ motivations [11,62] and personality traits [60].

Our work follows in the spirit of this theoretical framework, but with important differences in how we implement the overarching idea. Module (2) uses mental state representations inferred via inverse planning to generate probabilistic representations that reflect observers’ latent reasoning about how a target will appraise a situation. We refer to these latent representations as *computed appraisals*. Whereas *reverse* appraisals reflect an inverse inference (appraisals are inferred from their effects), *computed* appraisals reflect a forward inference (appraisals are computed from their causes). A related approach is seen in multi-agent partially observable Markov decision processes (POMDPs), where simulated agents compute appraisals based on their action planning policies and belief updates, and can infer appraisals of other agents by representing models of their states [12,23,24]. At present, these computer simulations have not been tested against human judgements.

Another related line of work has computationally modelled human observers’ emotion understanding as a Bayesian intuitive theory [3,4,55,63,64], see [13] for review. This line of research has not yet incorporated inverse planning. For example, Ong *et al.* [4] studied how observers predict others’ emotional reactions to a lottery. Observers’ predictions of eight emotions were well captured by a model based on three deterministically computed appraisal variables: the amount won, the reward prediction error and the absolute value of the reward prediction error. While groundbreaking, this prior model has a limited representational space. Players in a lottery make no decisions and have no social interactions, and the model predicts the same emotion for every player who received the same outcome. In another example, Wu *et al.* [55] used an inverse planning model to study how observers infer preferences and beliefs based on a person’s emotional reaction to events (e.g. if a person smiles at the outcome, she wanted it; if she opens her eye wide, she did not expect it), but not how observers predict emotions from inferred preferences and beliefs. In our framework, we therefore formalize the computation of appraisals using the machinery of inverse planning to reason over a Bayesian intuitive theory of emotion, combining key components of prior work.

### (c) Modelling emotion understanding

In our proposed framework, computed appraisals act as a latent mental grammar of emotion understanding, and emotion concepts reflect computations in this latent space [65]. Module (3) translates computed appraisals into emotion predictions by applying ‘emotion concept’ functions that define emotions in terms of computed appraisals. There are many possible ways to accomplish this step (essentially, writing a dictionary of emotion labels). It might be possible to constrain these definitions manually, by consulting formal and intuitive theories of the meanings of emotion labels [20,66–73]. In the present work, we preferred to learn the transformation from computed appraisals to emotions. Thus, our strategy can be situated within two tensions among modelling approaches.

The first tension relates to how reliant models are on humans. Computer science research tends to emphasize stimulus-computability, building and training models that operate over stimuli directly. Psychology research tends to emphasize the testing of psychological hypotheses, using human observers to abstract mental state representations from stimuli. The second tension relates to how model structure is acquired. Theory-driven approaches define representations and computations upfront. Data-driven approaches learn these from training data.

Some computer science work favours building highly structured causal models with cognitive latent spaces. This approach forgoes learning transformations between latent variables and emotions, opting to hand-code computations for appraisal variables that are prescribed by Appraisal Theories [12,23,24], see [18,74] for reviews. Appraisal variables are then translated into emotions according to prescribed definitions [22,75–77]. Other computer science work favours training less-structured models. This approach forgoes extensive hand-coding, opting to learn latent representations from statistical regularities in large datasets [78–80], see [81–83] for reviews. These learned latent representations can encode patterns mapping between emotion labels and expressions, scenes, objects, actions and social interactions [84–88].

Psychology research has aimed to build and test first- and third-person appraisal theories by modelling the relationships between empirical ratings of appraisals and of emotions. This approach relies on human observers to generate representations of stimuli. Observers judge someone’s appraisals and emotions based on information about the situation or the person’s expression, and emotion concept functions are fit or tested on these data [11,54,61]. Given manually annotated appraisal variables, simple classifiers can match human labels for many emotional events [54]. For example, a classifier given human ratings of 25 appraisal variables picked the self-reported emotion label (from 14 choices) for 51% of 6000 real-life events [68].

Our present work takes a middle path between these various approaches. We build a highly structured causal model based on theoretical constraints and psychological insights into social decision making, appraisal computations and emotion understanding. However, we implement general cognitive principals from Appraisal Theories, behavioural economics and computational cognitive science, rather than predefined representations of appraisal variables and emotion concepts. Primitive appraisal computations (achieved utility, prediction error, counterfactual reasoning) are applied over abstract Theory of Mind representations (preferences and beliefs), which are inferred from the event by inverting a causally structured generative model. The latent space of computed appraisals is then used to learn the conceptual structure of emotion predictions. Thus, we leverage probabilistic programming to infer, generate and discover the cognitive structure of the human intuitive theory of emotion.

Our formulation is situation-computable, operating over the same information given to observers. Since the model does not rely on humans to judge appraisals, the latent space of computed appraisals can be larger and more complex than empirically rated appraisal variables. In addition to reflecting sophisticated latent Theory of Mind reasoning, computed appraisal variables (and the learned emotion concepts) are interpretable owing to the cognitive structure of the generative model.

### (d) Comparing our model to human observers

In this paper, we compare our framework to human observers in three ways. First, we compare our model’s inverse planning against humans observing the same game. Second, we compare our model’s capacity to capture inferences of nuanced social emotions following outcomes of the games. Third, we test the model’s ability to adjust to personalizing information about individual players in these games.

The ‘Split or Steal’ game is socially rich, involving high-stakes social coordination, trust, betrayal, equity and public reputation. In the first set of experiments, we tested hypotheses about how observers reason about players’ action planning. Module (1) formalizes these hypotheses by inverting forward planning models to infer observers’ attributions of players’ preferences and beliefs. Empirical studies of social games make evident that players value not only money, but also how their actions will impact others [89–92]. In a one-shot social dilemma like the ‘Split or Steal’ game, an agent maximizing monetary payoff would always choose to defect [2]. By contrast, humans playing ‘Split or Steal’ chose to cooperate about half of the time [1], because they bring non-monetary social values into these games. We predicted that observers infer that players are motivated by social values, such as inequity aversion and reputational signalling; and observing a player’s choice to cooperate or defect leads people to update their understanding of the player’s preferences and beliefs. The results support these hypotheses, indicating that latent action planning representations are intuitive to observers and that the cognitive structure of the inverse planning model captures observers’ mental state inferences.

In the next set of experiments, we investigated the cognitive structure of emotion concepts. To sample the sophistication and breadth of observers’ intuitive reasoning about players in this game, we collected empirical predictions of 20 nuanced emotion labels, which we adapted for this task from prior work [54,68] (see the electronic supplementary methods §S1e). We hypothesized that capturing the empirical pattern of emotion predictions requires that a model represent observers’ intuitive reasoning about players’ decision-making and event appraisals. Module (2) formalizes hypotheses about how observers use representations of preferences and beliefs to reason about players’ event appraisals. Drawing from Appraisal Theories, we predicted that emotion judgements depend on specific computations (achieved utility, prediction error, counterfactuals) over inferred mental states. For example, emotions like *joy*/happy are functions of achieved utilities [4] and *fury*/rage are functions of prediction errors [68]. Emotions like *disappointment* depend on counterfactual reasoning about how events outside of a person’s direct control could have been different [93], and *embarrassment*/shame depend on counterfactual reasoning about how one’s own actions could have been different [94]. Module (3) learns transformations between computed appraisals and empirical emotion predictions. Lesions of these modules confirm that inverse planning is necessary to capture emotions that depend on inferences of why a player acted a certain way (not simply what events the player experienced), like *guilt*, and social appraisals are necessary to capture emotions that depend on interpersonal interactions, like *embarrassment* and *envy*.

Finally, a key empirical phenomenon is that individual people may react differently to the same outcome.^2^ Observers can use multiple sources of information to predict individual players' emotions. Because our model links prior beliefs, intentional actions, appraisals of subsequent events and emotion concepts, new information (e.g. different priors) propagates through the causal structure to update connected representations. We provided observers with personalizing information about specific players and collected preference and belief attributions and emotion predictions, for the individual players. Thus, our model can capture how observers update their emotion predictions based on personalizing information.

## 3. Inverse planning with social values

In our model of observers’ intuitive theory of emotion, inferred mental contents serve as the basis for computing appraisals and predicting emotions (figure 1). Module (1) implements a richly structured inverse planning model [15,17], which simulates how observers infer preferences and beliefs by reasoning over an intuitive theory of players’ minds. A forward model simulates what actions would be made by players with varying preferences and beliefs by formalizing causal relations between players’ mental contents and their behaviour. Bayesian model inversion then enables inference of the joint preferences and beliefs that a player was likely to have, given the player’s decision to cooperate or defect in the ‘Split or Steal’ game.

In the forward planning model, we incorporate social equity utilities that account for people’s actual decisions in social dilemmas [97,98]. Fehr & Schmidt [97] proposed that humans are motivated, to varying degrees, by two kinds of concerns for fairness in social interactions. Disadvantageous inequity aversion (*DIA*), a preference not to end up worse off than others, is a powerful and culturally conserved social preference [99,100]. In the context of ‘Split or Steal’, *DIA* is a preference not to be left with nothing while the other player steals the whole pot. In addition, Fehr and Schmidt observed that people’s choices reflect advantageous inequity aversion (*AIA*), a preference not to extract more than one’s fair share of a resource. In the context of ‘Split or Steal’, *AIA* is a preference not to steal the whole pot and leave the other player with nothing. Rational planning in the game thus maximizes utility over both non-social (monetary) and social (interpersonal inequity aversion) preferences, given expectations of the opponent’s choice.

We hypothesize that observers have an intuitive grasp of players’ social and monetary values. After observing a player’s choice, observers update their estimate of the player’s monetary and social values, and expectations. We begin by simulating players in an anonymous version of ‘Split or Steal’, and then extend the forward model to the actual public game.

### (a) Inverse planning in an anonymous game

We first simulated how players privately decide whether to cooperate (C) or defect (D) in an anonymous version of ‘Split or Steal’. In this AnonymousGame model, players have preferences exclusively for ‘base’ features, i.e. variables that can be objectively calculated from the situation and do not require mental state inference. We use the utility parametrization of Fehr & Schmidt [97] as our base features: player 1’s total monetary reward (Money), how much more player 1 received than player 2 (advantageous inequity, AI), and how much more player 2 received than player 1 (disadvantageous inequity, DI); see the electronic supplementary material, figure S2.

Payoffs to the players are determined by the action of player 1 ($a_{1}$), the action of player 2 ($a_{2}$) and the amount of money in the jackpot (pot). Thus, the outcome of a game is represented by the tuple $\left\langle a_{1},a_{2},pot\right\rangle$, and the base features are deterministic functions of this tuple.

We simulate player 1’s decision making as approximately rational subjective utility maximization over these base features. Simulated players are endowed with preferences and beliefs, which are randomly sampled from an empirically fit prior (see BasePrior in the electronic supplementary methods §S1j). Continuous preference weights (ω∈[0,1]) modulate the subjective utility that player 1 derives from the base features. A weighted expectation about what choice the opposing player will make ($\pi_{a_{2}}\in [0..1]$) models player 1’s latent belief about $\text{P}(a_{2})$. A prior belief about the expected value of the game ($\pi_{Money}\geq 0$) models how much money player 1 expected to win before the player learned how much money was actually in the pot.

A simulated player calculates the expected utilities of the action choices ($a_{1}\in \{\text{C},\text{D}\}$) based on its preferences and beliefs:

3.1 $$E\left[U^{\mathrm{base}}(a_{1})\right]=\sum_{a_{2}}\pi_{a_{2}}\cdot \left[\omega_{Money}^{\mathrm{base}}\cdot \nu (Money-\pi_{Money})-\omega_{AIA}^{\mathrm{base}}\cdot \nu (AI)-\omega_{DIA}^{\mathrm{base}}\cdot \nu (DI)\right].$$

The negative signs associated with AI and DI indicate that players seek to minimize inequity, thus $\omega_{AIA}^{\mathrm{base}}$ and $\omega_{DIA}^{\mathrm{base}}$ reflect a player’s advantageous and disadvantageous inequity *aversion*. The value function ν(⋅) and the reference point $\pi_{Money}$ reflect insights from prospect theory and the study of how people value uncertain rewards [101] (for details of implementation, see the electronic supplementary methods §S1f). Because pot sizes in the ‘Split or Steal’ game can range from $1 to over $100k USD, we adjust the utilities to reflect people’s nonlinear valuation of rewards. The value function (ν) applies a transformation commonly used in behavioural economics to account for people’s diminishing marginal utility [102,103]. For our purposes, ν amounts to a sign-adjusted logarithm that treats gains and losses symmetrically. Because players on the ‘Split or Steal’ gameshow, and observers in our studies, know the range of pot sizes before the game begins, we model players as having an expectation about how much they would win. The reference point $\pi_{Money}$ adjusts monetary utility relative to how much a simulated player expected to win before learning the pot size. Rewards that fall short of the reference point are perceived as negative utilities.

To calculate the expected utility of an action ($a_{1}$), simulated players integrate subjective utility over the opposing player’s possible actions ($a_{2}\in \{\text{C},\text{D}\}$). This involves scaling the subjective utility of an outcome by player 1’s belief that player 2 will choose $a_{2}$. The expected utility of a simulated player choosing action $a_{1}$ is $E[U^{\mathrm{base}}(a_{1})]$. Decision making follows probabilistically by sampling from the softmax of expected utility: $P(a_{1}|\omega^{\mathrm{base}},\pi_{a_{2}})\propto \exp(\lambda \;\cdot \;E[U^{\mathrm{base}}(a_{1})])$.

The softmax function is a standard decision policy for modelling an agent’s planning and decision-making in uncertain environments [104] and for observers’ reasoning about others’ noisy choices [15,16,36,38,40]. The thermodynamic parameter λ determines how rationally versus noisily decisions reflect differences between the expected utilities of choices considered by a simulated player. We marginalize over $\pi_{Money}$ since we did not collect empirical attributions or fit the prior.

The AnonymousGame forward planning model can be inverted to infer observers’ attributions of mental contents to players in an anonymous version of the ‘Split or Steal’ game (see the electronic supplementary material, figure S3 for empirical attributions and model inference of preference and belief weights). However, as a model of the ‘Split or Steal’ game, the AnonymousGame model is incomplete. To serve as a basis for emotion prediction for the public game, we needed to introduce a salient aspect of the Golden Balls gameshow: the audience.

### (b) Second-order preferences: players’ motives for reputation

The AnonymousGame model is missing a critical element of social strategy games: players’ motive to enhance their reputation. For example, Arthur may choose to cooperate primarily to signal his cooperativeness to future social partners. We hypothesize that human observers can infer such second-order preferences, and that inferences about the motive to enhance one’s reputation underlie prediction of key social emotions, like *embarrassment* and *pride* [105]. We therefore extended the generative model of decision planning in an anonymous game to include players’ reputation concerns.

A standard way to incorporate reputation concerns might be to add additional utility variables that define the reputational consequences players expect of their actions. Unlike the base features, the reputation signals cannot be objectively computed directly from the events and must therefore be specified for each situation and action. We follow a more cognitively natural strategy, whereby players apply their own Theory of Mind to anticipate how others will evaluate them [36]. To choose an action that is reputation enhancing, players must first infer how their behaviour will be interpreted by others. This requires an embedded inference loop. We model a simulated player’s expected reputation as the inferences a rational observer would make about the weights of the player’s base utility function ($\omega^{\mathrm{base}}$). The inferred base weights are themselves weighted and treated as ‘second-order’ utilities. Thus, a simulated player’s expected reputation reflects the player’s belief, and preference, about what other people will think the player’s values are.

In the PublicGame model, we introduce a reputation utility for each base feature. The expected reputational consequences of a player’s action are weighted by randomly sampled preferences ($\omega^{\mathrm{repu}}$), which model how much the player cares about other people’s beliefs. The expected utility of an action is the sum of the expected base utilities and the expected reputation utilities:

3.2 $$\begin{array}{rl} \text{E}[U^{\text{base}+\text{repu}}(a_{1})] & \;=\text{E}[U^{\text{base}}(a_{1})]-\omega_{Money}^{\text{repu}}\cdot \nu (\text{E}[\omega_{Money}^{\text{base}}|a_{1}]\cdot pot)\\ & \;\;+\omega_{AIA}^{\text{repu}}\cdot \nu (\text{E}[\omega_{AIA}^{\text{base}}|a_{1}]\;\cdot \;pot)\\ & \;\;+\omega_{DIA}^{\text{repu}}\cdot \nu (\text{E}[\omega_{DIA}^{\text{base}}|a_{1}]\;\cdot \;pot), \end{array}$$

where $\text{E}[\omega^{\mathrm{base}}|a_{1}]$ is a simulated player’s expectation of what other people would infer about the player’s base preference, if the player chooses $a_{1}$. The expected base utility of an action, $\text{E}[U^{\mathrm{base}}(a_{1})]$, is the same as in the AnonymousGame model, equation (3.1). The sign on each reputation utility is opposite that of the corresponding base utility, reflecting the hypothesis that observers believe that players desire to be seen as motivated to improve equality, and not motivated to selfishly maximize their own monetary payoffs. We assume that reputation utilities are functions of the pot size (the higher the stakes of a decision, the more consequential the reputation signals).

A simulated player calculates the expected utility of an action, $\text{E}[U^{\mathrm{base}+\mathrm{repu}}(a_{1})]$, based on randomly sampled base preferences ($\omega^{\mathrm{base}}$), reputation preferences ($\omega^{\mathrm{repu}}$), belief about which action the opponent is likely to choose ($\pi_{a_{2}}$), prior belief about the expected value of playing the game ($\pi_{Money}$) and beliefs about the inferences other people will make of the player’s base preferences ($\text{E}[\omega^{\mathrm{base}}|a_{1}]$). Decision making follows probabilistically by sampling from the softmax of expected utility:

3.3 $$P(a_{1}|\omega^{\text{base}},\omega^{\text{repu}},\pi_{a_{2}})\propto \exp(\lambda \;\cdot \;\text{E}[U^{\text{base}+\text{repu}}(a_{1})]).$$

Preferences, and the belief about the opponent’s action, are sampled from an empirical prior (see GenericPrior in the electronic supplementary methods §S1j).

The purpose of building a generative model that simulates how players with varying preferences and beliefs make decisions in the ‘Split or Steal’ game, is to capture observers’ latent reasoning about players’ intentional actions. Namely: What were a player’s mental contents given that the player chose to cooperate or chose to defect? Since the forward model of decision making is invertible, it supports inverse inference of a players’ preference and belief weights. We invert the PublicGame forward model using Bayes’s rule:

3.4 $$\text{P}(\omega ,\pi_{a_{2}}|a_{1})\propto \text{P}(a_{1}|\omega ,\pi_{a_{2}})\;\cdot \;\text{P}(\omega ,\pi_{a_{2}}),$$

where ω is the six-element vector of base and reputation preference weights. $\text{P}(\omega ,\pi_{a_{2}}|a_{1})$ is the joint posterior distribution over preference and belief weights conditional on player 1’s action.

### (c) Comparison to human observers

The first goal of our model is to capture the inferences observers make about players’ preferences and beliefs. We therefore tested whether human observers systematically infer the players’ values and expectations from observing a single choice, and whether we could capture these inferences by inverting our generative model of players’ behaviour. We presented Amazon mTurk participants with scenarios depicting one player’s decision to cooperate or defect on the ‘Split or Steal’ gameshow. These scenarios were synthesized from the range of possible pots and decisions, rather than reflecting any actual recorded episode. The observers judged players’ base and reputation preferences, and belief about the other player’s intended decision (see electronic supplementary methods §S1a for details of the data collection).

**Figure 2.**
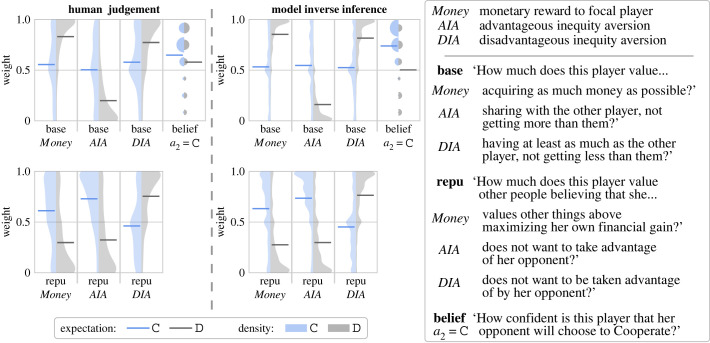
Inverse planning. Human observers were shown a player’s decision to cooperate (C) or defect (D) and judged the player’s likely preferences and belief. Model inversion yields a joint inference of the mental contents conditional on the players’ actions. Preference weights take continuous values between zero and one. Player 1’s belief about what player 2 will choose was rated on a 6-point confidence scale. Expectation shows the mean weight of each marginal distribution conditional on the player’s action ($a_{1}$).

We found that human observers readily and consistently inferred the psychological features from under-specified input. Observers’ judgements and the inverse inference of the PublicGame model are shown in figure 2. Human observers and the model made very similar inferences from the same observed actions. For example, observers inferred that Arthur’s (player 1’s) decision to cooperate means that he was likely to be less motivated by acquiring as much money as possible (base Money), more averse to gaining an unequal and superior outcome (base AIA), and less adverse to receiving an inferior outcome (base DIA), than if he had defected. If Arthur cooperated, he was also judged to care more about people believing that he values other things above maximizing his own financial gain (repu Money) and does not want to take advantage of her opponent (repu AIA). If he defected, he was judged to care more about people believing that he is a strong competitor and not one easily taken advantage of (repu DIA).

In sum, the PublicGame forward planning model can generate plausible player choices, and can be inverted to make inferences about players’ values from the observation of a single choice. Observers make similar systematic inferences of players’ values from the same observation. Both observers and our model successfully resolve an ill-posed inverse problem, to recover latent mental contents that motivated players’ behaviour.

As a generative model of decision making, the PublicGame model is much richer than is necessary to predict players’ choices in a Prisoner’s Dilemma, which can be captured by extremely simple models [106]. Nevertheless, our interest is in what observers infer about the latent mental contents that underlie the decisions made by others. For this purpose, the richer model better captures the inferences human observers make and supports the subsequent inference of players’ fine-grained reactions. Most importantly for our current purposes, we expect that this richness is necessary to capture the predictions that observers make about players’ emotions.

## 4. Computed appraisals

Through the successive inversion of increasingly rich generative models of behaviour, we have built an inverse planning model that uses a player’s choice in a social game to infer the joint posterior probability of the player’s selfish, social and reputational preferences, and belief about the opposing player’s intended action. The preferences and beliefs inferred by Module (1) serve as the basis for computing appraisals in Module (2) (figure 1*b*). Computed appraisals are latent Theory of Mind representations: how players evaluate a new world state (the outcome of the game) caused in part by an event outside of the player’s intentional control (the opponent’s action) given the player’s mental contents.

As the cognitive latent space for predicting emotions, computed appraisals need to represent the computational structure of observers’ emotion concepts. Drawing from prior work, we define primitive appraisal computations, which are applied over inverse planning representations. We implement four types of appraisal computations based on achieved utility (AU), utility prediction error (PE) and counterfactual utilities with respect to the actions of player 1 and player 2 (${CFa}_{1}$ and ${CFa}_{2}$, respectively). The rest of this section explains how these appraisals work in high-level terms; formal definitions are given in the electronic supplementary methods §S1i.

**Figure 3.**
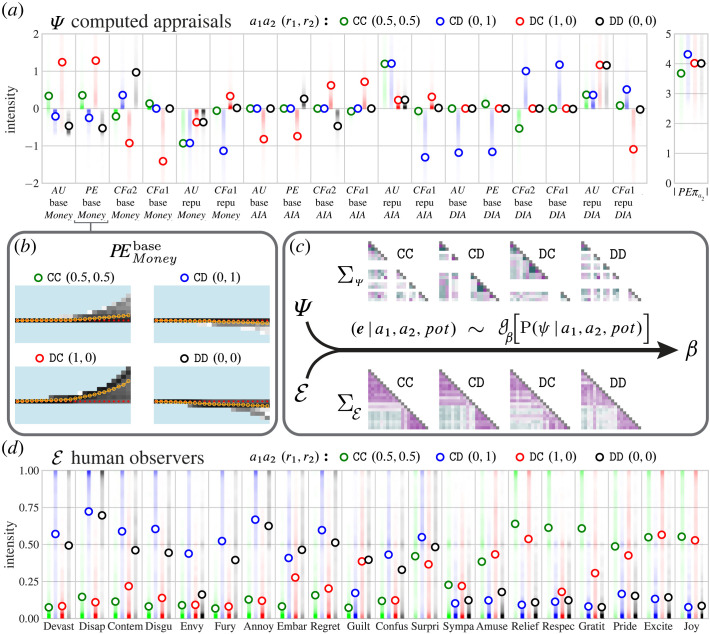
Generative structure. (*a*) Expectations and densities of the normalized computed appraisals. Ψ is the matrix of the 19-dimensional appraisal vectors. The legend gives the relative payoffs to the players ($r_{1},r_{2}$), e.g. when player 1 Cooperated and won nothing because player 2 Defected and won the whole pot, $a_{1}a_{2}\;(r_{1},r_{2})$: CD (0,1). Colour indicates the outcome of the games. (*b*) Example of a computed appraisal’s relationship to pot size. The x-axis shows the 24 pot sizes (non-parametric scaling), y-axis shows the loading on simulated players’ monetary utility prediction error, colour indicates density. (*c*) We learn a function (g) that transforms computed appraisals (Ψ) into emotion predictions (E) by scoring the empirical emotion vectors predicted for the *GenericPlayers* from the joint posterior over computed appraisals. To learn the transformation parameters, we leverage the expectations, as well as the hierarchical covariance structure of computed appraisals and of empirical emotion attributions. The result is a sparse weights matrix β. Expectations are shown in (*a*,*d*). Correlation matrices shown in (*c*) give the within-stimulus correlation (averaged within outcome) between computed appraisals (top) and between empirical emotion predictions (bottom). (*d*) Emotion predictions for the *GenericPlayers*. Circles show the expected intensity for each outcome, summing over pot sizes and the eight photos. Shading shows the density of judgements. E is the matrix of the 20-dimensional emotion prediction vectors. Each expectation (and associated distribution) reflects n=1 108 judgements of n=554 observers.

In the PublicGame model, players make decisions by calculating the expected utility of their actions. When the opponent’s action is revealed, and the outcome of the game is known, simulated players learn the utilities they achieved (AU) and the error signals between these outcomes and what they expected (PE). The same mental contents that led a simulated player to choose an action can be used to compute the utilities that *would* have been achieved, if the player had made a different choice (${CFa}_{1}$), or if the opponent had made a different choice (${CFa}_{2}$). We leverage these rich mental state representations to compute appraisals over monetary, social and reputational representations, incorporating the simulated player’s beliefs about what was going to happen, what did and did not happen, and how the player and the opponent could have changed what happened. Figure 3*a* shows how the events of the ‘Split or Steal’ game load onto the computed appraisals.

Computed appraisals follow a similar nomenclature as utilities in §3, defined in terms of ‘*Money*’, ‘*AIA*’ and ‘*DIA*’ utility terms, and both first-order (‘base’) and second-order (‘repu’) preferences. To illustrate more concretely how our four types of appraisals are defined, consider just the base monetary reward. During planning, the base monetary utility a player expected is written as $\text{E}U_{Money}^{\mathrm{base}}$. When the player learns the opponent’s decision (and correspondingly, the outcome of the game), the same mental contents that led to the player’s choice determine the subjective utility that the player achieves. Thus, the achieved utility (AU) for base monetary utility, written as ${AU}_{Money}^{\mathrm{base}}$, reflects the simulated player’s subjective valuation of the event based on the player’s preference ($\omega_{Money}^{\mathrm{base}}$) and how much Money the event confers.

Prediction error (or ‘expectation violation’) is the discrepancy between what was expected and what occurred. Extensive research illustrates that prediction error is a fundamental computation in first-person emotion experience [107–113] and in third-person emotion understanding [13,114] of emotions like *surprise*, *disappointment* and *fury*/rage [4,68,115]. For a simulated player’s base utility terms, we compute prediction error (PE) from the difference between the achieved utility and expected utility. In addition, we use a player’s weighted belief to compute the absolute prediction error of the opponent’s action (how unexpected the opponent’s behaviour was): $|PE\;\pi_{a_{2}}|$.

Counterfactuals involve mental simulations of alternative realities, and are central to causal reasoning. For our current purposes, it is comparing what could have happened to what did happen. Counterfactual judgements are a fundamental computation in first-person experience [19,116–119] and in third-person emotion understanding [4,63,120–123]. Reasoning about counterfactual events outside of a person’s direct control is implicated in emotions like *disappointment* and *relief*, while reasoning about how a person could have changed the situation by choosing a counterfactual action is implicated in emotions like *embarassement*, *regret* and *guilt* [93,94,124,125].

We compute counterfactual contrasts based on the action not chosen by each player. For the opponent’s action counterfactual (${CFa}_{2}$), which a simulated player cannot directly control, the contrast between the counterfactual utility and achieved utility is weighted by the simulated player’s belief that the opponent would make the other choice: $\pi_{\neg a_{2}}$. For the simulated player’s action counterfactual (${CFa}_{1}$), the contrast is weighted by the probability that the player would have made the other choice, given the player’s preferences and updated belief about the opponent’s action^3^: $\text{P}(\neg a_{1}|\omega ,a_{2})$.

In sum, the machinery of inverse planning makes it feasible to compute probabilistic representations of players’ appraisals. The latent space of computed appraisals reflects sophisticated causal reasoning about recursive social preferences and beliefs. We now leverage these abstract Theory of Mind representations to reverse-engineer the cognitive structure of observers’ emotion concepts. In effect, we learn a Computational Appraisal Theory directly from observers’ emotion judgements.

## 5. Emotion predictions

We now return to the challenge that we began with: testing whether our computational model can capture the conceptual knowledge and intuitive reasoning that underlie human observers’ emotion predictions.

### (a) Human observers’ emotion predictions

We collected human observers’ predictions of the emotions players would experience when the outcome was revealed in ‘Split or Steal’ games. We collected two datasets from online participants. The training data (n=554) were used to learn a transformation between the latent space of computed appraisals and the emotion predictions, which was then used to predict emotions for the test data (n=1 512). In the test data, observers were presented with specific information about each focal player. Collection of these data, referred to as the *SpecificPlayers*, will be described in §6.

In the training data (*GenericPlayers*), observers were briefed on the structure of the ‘Split or Steal’ game and watched a video taken from the show in which the presenter explains the rules and two players negotiate in an attempt to convince the other to choose ‘Split’ (*cheap talk* negotiation). The introductory video ends before the players reveal their choices. Observers completed eight trials, in which they saw a photograph of the focal player (designated player 1), a pot size (ranging from $2 to $207365 USD), and the actions chosen by both players in that game. Observers saw two games for each category of payoff: CC where both players cooperated and each won half; CD where player 1 cooperated and received nothing; DC where player 1 defected and took everything; and DD where both players defected and both got nothing. Observers then predicted how much player 1 would experience 20 different emotions: *Devastation*, *Disappointment*, *Contempt*, *Disgust*, *Envy*, *Fury*, *Annoyance*, *Embarrassment*, *Regret*, *Guilt*, *Confusion*, *Surprise*, *Sympathy*, *Amusement*, *Relief*, *Respect*, *Gratitude*, *Pride*, *Excitement* and *Joy*.

To learn a transformation between the model and human emotion judgements, we make use of the rich structure present in the observers’ emotion predictions. The *GenericPlayers* data (figure 3*d*) illustrate that, even from sparse event depictions, human observers made systematically different emotion predictions for players in the four different outcome categories. At the coarsest qualitative level, observers predicted that players who won money (CC and DC outcomes) would experience more positive emotions and players leaving with nothing (CD and DD outcomes) would experience more negative emotions (figure 3*d*). However, observers’ emotion predictions do not just reflect the monetary outcomes of the game. For example, when player 2 defects, player 1 necessarily receives no monetary reward, yet observers predicted that player 1 would have different emotional reactions depending on whether he chose to cooperate (more *envy* and *contempt*) versus defect (more *guilt*). Note that preference/belief attributions (described in §3) and emotion predictions were collected from mutually exclusive groups to avoid cueing observers to think about emotion experience in terms of the planning variables or vice versa.

### (b) Learning the latent structure of the intuitive theory of emotion

We hypothesized that human observers infer players’ values and expectations from their actions using inverse planning, and predict players’ emotional reactions to events based on those inferred mental states. In this model, emotion predictions reflect observers’ reasoning about how players would react to an event given the particular players' beliefs and preferences. Critically, we assume that emotion prediction relies on inverse planning. Inferred mental contents, generated via the inversion of an intuitive Theory of Mind, form the basis for reasoning about how a player will evaluate events. In our proposed model of the intuitive theory of emotion (figure 1), computed appraisals serve the functional role of linking observations (the events in a ‘Split or Steal’ game) and emotion concepts. Thus we call our model of emotion prediction based on mental states inferred from inverse planning, a ComputedAppraisals model.

In Module (1), the model uses the pot size and player 1’s chosen action to update estimates of player 1’s preferences and beliefs. In Module (2), these preferences and beliefs then affect how the player appraises the situation. To generate computed appraisals for the *GenericPlayers*, we ran the PublicGame model in the same way as in §3b: using an empirically derived prior over the base preferences and beliefs, we inverted the hierarchical generative model of behaviour. Then, we computed how simulated players would appraise the outcome of the game. Appraisals are computed as the achieved utilities, prediction errors and counterfactuals, on a player’s beliefs, base preferences and reputation preferences. See the electronic supplementary methods §S1i for details of how appraisals are computed, and the electronic supplementary methods §S1j for details about the priors. Module (3) transforms computed appraisals into emotion predictions by applying learned emotion concepts: functions that translate appraisal loading into emotion intensities. Thus, before we can generate emotion predictions, we need to learn the ‘meaning’ of each of the 20 emotion labels that human observers rated, in terms of the set of appraisal variables.

To learn the function relating emotion labels to computed appraisals, we made a strong assumption about the generative structure of observers’ emotion predictions: when people are asked to predict a player’s emotions, they do not make 20 independent inferences but rather infer a joint distribution over the player’s preferences and beliefs, and reason about how these inferred mental contents would cause the player to evaluate the situation (figure 1). For instance, if player 1 cooperated while player 2 defected (CD), how much an observer thinks that player 1 wanted to avoid being disadvantaged will be reflected in that observer’s predictions of player 1’s experience of both *embarrassment* and *envy*. Thus, the covariance patterns of observers’ emotion predictions reflect the latent structure of their intuitive theory of psychology. Using the *GenericPlayers* training data, we leverage this information to learn a mapping between people’s empirical emotion predictions and the joint distribution over appraisal variables generated by the model.

Figure 3 shows the model’s computed appraisals and observers’ emotion predictions for the *GenericPlayers*. Figure 3*a*,*d* show the expected values given an outcome. Figure 3*c* shows the average within-stimulus correlation matrix for each outcome. Note that formalizing computed appraisals as a probabilistic generative model permits us to leverage within-stimulus covariance in latent structure discovery.

Based on the *GenericPlayers* data shown in figure 3, we learn a sparse transformation between the joint distribution of computed appraisals and the joint distribution of emotion predictions. Specifically, we treat the empirical emotion prediction vectors as observations from some function of the posterior distribution of computed appraisals (g in figure 3*c*). We find a transformation of the appraisal distribution that maximizes the probability of observing the empirical data under a Laplace prior on the transformation coefficients. This yields a sparse transformation between computed appraisals sampled from the ComputedAppraisals model and continuous quantitative predictions of the player’s emotions.

**Figure 4.**
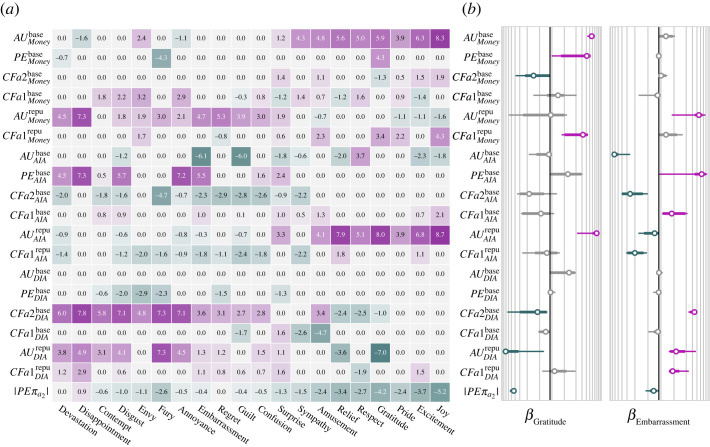
Appraisal structure of the intuitive theory of emotion. The β weights of the transformation were learned based on the joint distribution of appraisals and the joint distribution of emotion predictions for the *GenericPlayers*. A Laplace prior over the β weights induces a sparse solution. To learn the scale of the prior, we cross-validated on subsets of the *SpecificPlayers* and generated emotion predictions for the leftout players. Saturation indicates that the 99% CI does not overlap with zero. (*a*) Mean weights of the learned transformation. (*b*) These log-scale plots show the expectation, 95% and 99% CI of the weights learned for two example emotions, *gratitude* and *embarrassment*.

This model recapitulates the social cognitive reasoning that allows human observers to predict players’ emotions for arbitrary games. The emotion concepts shown in figure 4 reflect a computational hypothesis about the intuitive theory of emotion. This hypothesis says that observers’ emotion predictions reflect latent computations over players’ inferred mental contents. For example, a player who is inferred to value money highly will experience more *gratitude* when she wins more money $\left({AU}_{Money}^{\mathrm{base}}\right)$, and when she wins more than she expected $\left({PE}_{Money}^{\mathrm{base}}\right)$. A player will experience more *gratitude* when her opponent could have prevented her from winning money but chose not to, and less *gratitude* when her opponent could have chosen an action that would have caused the player to win money but instead chose an action that resulted in the player winning nothing $\left({CFa_{2}}_{Money}^{\mathrm{base}}\right)$. The more a player cares about not being in a socially disadvantageous position the more *gratitude* she will experience when her opponent could have taken advantage of her but chose not to, and the less *gratitude* she will experience when her opponent decided to exploit her $\left({CFa_{2}}_{DIA}^{\mathrm{base}}\right)$. The more a player is motivated to be seen as a fierce competitor, the less *gratitude* she will experience $\left({AU}_{DIA}^{\mathrm{repu}}\right)$.

The appraisal pattern is quantitatively and categorically unique for each emotion, suggesting that these 20 emotions are conceptually distinct. Much of the learned structure qualitatively aligns with existing emotion taxonomies [68,72,126]. We next test if the computational hypothesis formalized by the ComputedAppraisals model captures human emotion predictions.

### (c) Comparing the computed appraisals model to human observers

**Figure 5.**
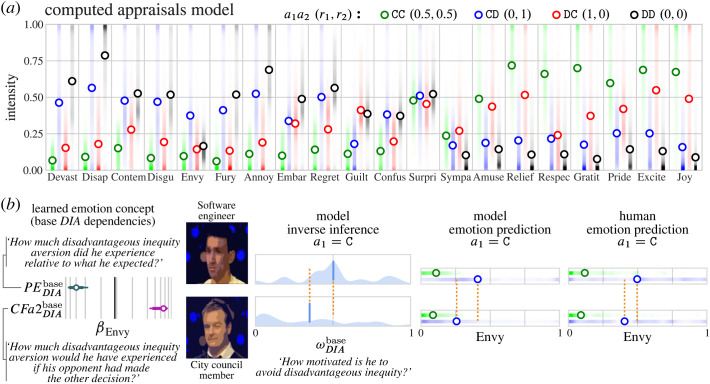
Inferred emotion predictions. (*a*) Emotion predictions generated by the ComputedAppraisals model, averaged across players and pot sizes. (*b*) Example of how the model personalizes emotion predictions. Based on the *GenericPlayers* data, the model learned that *envy* is a function of appraisals derived from a player’s aversion to disadvantageous inequity (base DIA). When a player appraises that he is in a more socially disadvantageous position than he expected to be (negative prediction error), the negative loading on $PE_{DIA}^{\mathrm{base}}$ translates to greater *envy* intensity. Similarly, when a player appraises that he would have been in a less disadvantageous position if his opponent had made the other choice (positive counterfactual), the positive loading on $CFa2_{DIA}^{\mathrm{base}}$ translates to greater *envy* intensity. Given that they chose to cooperate ($a_{1}=\text{C}$), the model infers that the engineer cares more about not ending up in an inferior position ($\omega_{DIA}^{\mathrm{base}}$) than the councilman. When the opposing player defects ($a_{2}=\text{D}$), the model predicts greater intensity of *envy* for the engineer because he is inferred to have a stronger preference. Human observers similarly predicted that the engineer will experience more *envy* than the councilman in CD games. Photos of the players have been downsampled for the purpose of publication.

The ComputedAppraisals model generates a joint distribution over 20 emotions based on the two players’ actions and the pot size $\left\langle a_{1},a_{2},\mathrm{pot}\right\rangle$ by generating computed appraisals under a prior distribution of player 1’s preferences and belief $\text{P}(\omega ,\pi_{a_{2}})$. Using the transformation, we learned based on the *GenericPlayers*, we generated personalized emotion predictions for 20 *SpecificPlayers*. The *SpecificPlayers* are described in detail in §6, but before considering how personalizing information biases emotion predictions for individual players, we first consider the overall structure of the model. The ComputedAppraisals model captures the overall pattern of human emotion judgements. Emotion predictions generated by the model (averaged over players and pot sizes) are shown in figure 5*a*. Positive emotions are predicted when players win money and negative emotions when players lose money. In addition, the model captures some of the more nuanced features of the empirical judgements. When a player’s opponent defects, causing the player to leave with no money, the model (like human observers) predicts more *envy* if the player cooperated (CD) and more *regret* if the player defected (DD). When the opponent cooperates, causing the player to win money, the model (like human observers) predicts more *relief* and *gratitude* if the player cooperated (CC) and more *guilt* if the player defected (DC).

**Figure 6.**
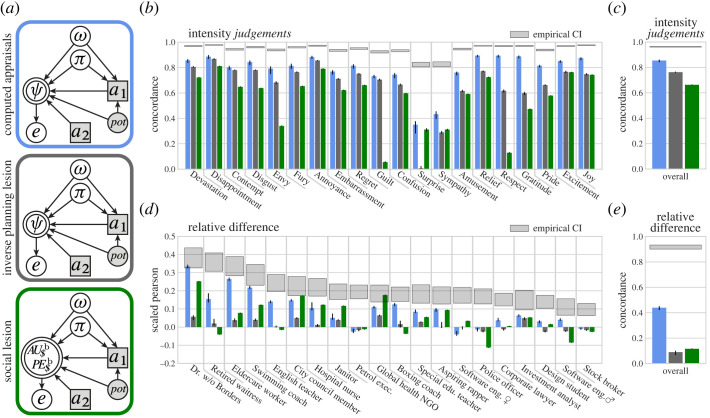
Predicting specific player’s emotions. Human observers made preference and belief attributions to the 20 *SpecificPlayers*, based on a photo, brief description and decision, in the ‘Split or Steal’ game. (*a*) Based on what a *SpecificPlayer* was judged to care about and to expect, the models generated predictions of that player’s emotion reaction in 24 ‘Split or Steal’ games (four outcomes and eight pots). Bar colours in (*b*–*e*) correspond to the models in (*a*), and grey windows give the 95% bootstrap CI of the inter-rater reliability of the emotion predictions. (*b*) Concordance between predictions generated by the models and human observers for every emotion (collapsing across players, outcomes and pot sizes). (*c*) Overall fit the emotions observers predicted for the 20 *SpecificPlayers*. (*d*) The photos and descriptions of *SpecificPlayers* biased human observers’ judgements of the players’ motivations, expectations and emotional reactions. This plot shows how well the models were able to predict the bias in emotion predictions based on observers’ judgements of a player’s preferences and belief. Players are ordered based on how reliably observers’ emotion predictions differed from the emotions predicted for the *GenericPlayers* (grey windows). The model score gives the variance-scaled Pearson correlation. (*e*) Correlation between the relative difference predicted by the models and the relative difference in observers’ emotion predictions. (*b*,*c*,*e*) Each bar reflects a model’s performance based on n=12 096 emotion predictions of n=1 512 observers. (d) Each bar reflects a model’s performance based on a minimum of n=579 empirical predictions of all 20 emotions.

To assess the explanatory power of the model, we use Lin’s Concordance Correlation Coefficient^4^ (ccc), which is a metric of the agreement between model predictions and a ground truth measure (the empirical emotion predictions) [127], and bootstrap resampling to estimate 95% confidence intervals (CI). Across all players, emotions, outcomes and pot sizes, the ComputedAppraisals model fit the observer’s emotion predictions for the *SpecificPlayers* data well: ccc=0.854 [0.844,0.859] (figure 6*c*).

Predictions of different emotions depend on different types of information, making it likely that a model’s latent representations will enable it to capture some emotions better than others. Similarly, human observers can find a stimulus ambiguous with regard to one emotion but unambiguous with regard to another (figure 6*b* shows the reliably of observers’ predictions and how well the ComputedAppraisals model captures the empirical emotion predictions for the *SpecificPlayers*). To test whether the rich generative structure of the ComputedAppraisals model significantly contributed to its ability to capture observers’ emotion predictions in this task, we compared the ComputedAppraisals model with two simpler alternatives.

### (d) Inverse planning lesion model

The InversePlanningLesion (figure 6*a*) selectively blocks inverse planning by inferring appraisal variables based on the prior distribution of beliefs and preferences (before either player acts), rather than the posterior distribution (updated based on the decision of player 1). Players simulated by the InversePlanningLesion model are effectively forced to choose a ball at random, without looking inside. Without a causal link to behaviour, the inverse inference of players’ preferences and beliefs reduces to the prior. Thus, the posterior probability in equation (3.4) becomes: $\text{P}(\omega ,\pi_{a_{2}}|a_{1})=\text{P}(\omega ,\pi_{a_{2}})$. We similarly lesion the embedded inverse planning loop, which simulates how their behaviour will be interpreted by others. Appraisal generation is identical to the ComputedAppraisals model and still depends heavily on $a_{1}$.

To illustrate the InversePlanningLesion, consider the effect of simulated players’ beliefs about their opponents’ actions ($\pi_{a_{2}}$) on the appraisals made by each model. In the full ComputedAppraisals model, as for human observers, simulated players only tend to cooperate when they believe their opponent is also going to cooperate (see $\text{E}[\pi_{a_{2}}|a_{1}=\text{C}]$ in figure 2). Players simulated by the InversePlanningLesion model select balls randomly, so the expectation of monetary utility reflects the prior on belief, $\text{P}(\pi_{a_{2}})$, rather than what beliefs were likely given the player’s action, $\text{P}(\pi_{a_{2}}|a_{1})$.

Across all players, combinations of decisions and pot sizes, the InversePlanningLesion model showed a lower fit to human observers’ emotion predictions (ccc=0.762 [0.760,0.764]; figure 6*c*). The effect of lesioning inverse planning is particularly evident in specific emotions. For instance, the InversePlanningLesion caused notable decrements in the capture of *envy*, *surprise*, *relief*, *gratitude*, *pride* and *joy* (figure 6*b*).

### (e) Social lesion model

In the SocialLesion model, we removed all of the social (non-monetary) values attributed to players. This lesion allows us to test the importance of these social values for successfully generating human-like emotion predictions. The SocialLesion model leaves forward-planning intact: simulated behaviour, inferred monetary utility and prediction error, are all identical to the full ComputedAppraisals model (figure 6*a*). The social lesion can be likened to observers having the intuitive theory that players’ emotional reactions depend only on monetary considerations. The SocialLesion model predicts 20 emotions from the transformation of the joint distribution of monetary utility and monetary prediction error, $\text{P}({AU}_{Money}^{\mathrm{base}},{PE}_{Money}^{\mathrm{base}}|a_{1},a_{2},pot)$.

Across all players, combinations of decisions and pot sizes, the SocialLesion model showed a lower fit to human observers’ emotion predictions (ccc=0.663 [0.663,0.663]; figure 6*c*). As expected, the SocialLesion model was largely unable to capture predictions of social emotions like *envy*, *guilt*, *gratitude* and *respect*. Interestingly, the SocialLesion model also provided a poor fit for predictions of some emotions that were well fit by monetary appraisals of lottery outcomes [4]. For example, observers’ predictions of players’ *joy* are positively related to monetary payoff in both lotteries and ‘Split or Steal’: for a given action, players who win $12k USD are predicted to experience more *joy* than players who win $6k USD. However, for a given pot size in ‘Split or Steal’, observers predict similar levels of *joy* for players who cooperated and took half the pot, as for those who defected and took twice as much. Human observers predict that *joy* reflects social values. With access to only monetary appraisals, the SocialLesion can capture the positive relationship between *joy* and the pot size, but not the way in which predictions of *joy* depend on the social consequences of an action.

## 6. Personalizing emotion predictions

So far, we have investigated how human observers, and the ComputedAppraisals model, predict emotional reactions for players after observing only a single action in the game. However, the structure of the game means that single actions are highly ambiguous. An observer who personally knows a specific player might be able to use prior knowledge, from outside the game, to inform inferences about the player’s likely values and expectations [128,129]. If the ComputedAppraisals model is a good approximation of how human observers reason about players’ emotions, it should also be able to predict the emotions observers predict *specific* players will experience.

To mimic prior knowledge of the players, we constructed 20 *SpecificPlayers*, each composed of a unique headshot and brief description. The descriptions included, ‘Doctor, volunteering in South Africa with *Doctors Without Borders*’, ‘Software engineer at Google’, and ‘City council member, about to start campaigning for State Senate’. We hypothesized that even such sparse information would evoke stereotypes that allow human observers to update priors over the players’ likely preferences and beliefs.

To test this hypothesis, we asked human observers to rate how much each *SpecificPlayer* actually valued, and valued others believing that they valued, *Money*, *AIA* and *DIA*, and what the players predicted their opponents would do, given the players’ decisions in ‘Split or Steal’. After being familiarized with the ‘Split or Steal’ game, each observer made preference and belief attributions to eight players. In each trial, observers were shown a player’s description (a photo and career), the player’s choice and the pot size. Confirming our hypothesis, human observers made consistent and distinct preference and belief attributions to *SpecificPlayers*, which differ from the attributions made to the unspecified *GenericPlayers*. For example, the software engineer is viewed as more motivated to avoid being taken advantage of than the city councilman, even when both were shown to have cooperated (figure 5*b*). The overall patterns of emotions observers predicted for the *GenericPlayers* were replicated by the emotion predictions made for the *SpecificPlayers* in this experiment. No player is expected to experience more *envy* after winning money than not winning, for example, but how much *envy* observers expect a player to experience differs between players. See the electronic supplementary material §S6 for a complete example.

### (a) Simulation of the bias induced by personalizing cues

If a model has learned an accurate mapping from computed appraisals to emotion judgements, then it should be sensitive to variation in the psychological characteristics attributed to specific players, which are the bases for computing appraisals. The key generalization test is therefore whether the ComputedAppraisals model accurately predicts how emotion predictions will differ between players in the same situation, based on observers’ ratings of each player’s preferences and beliefs.

The empirical expected difference between emotions predicted for a *SpecificPlayer* and the *GenericPlayers* is given by $\Delta e_{player}=\langle \delta_{\text{CC}}^{\;devast.},\delta_{\text{CD}}^{\;devast.},\ldots ,\delta_{\text{DD}}^{\;joy}\rangle$ where,

$$\delta_{\text{DD}}^{\;joy}=\text{E}[\;joy|\text{DD};player]-\text{E}[\;joy|\text{DD};generic].$$

In similar manner, the expected difference predicted by a model, given by $\Delta {\hat{e}}_{player}$, is the difference between the emotions a model predicted for a *SpecificPlayer* and for the *GenericPlayers*. Since this difference is calculated relative to a model’s own prediction of the *GenericPlayers* emotions, a model that fails to fit the absolute expected emotion intensities can still capture how observers’ emotion predictions for *SpecificPlayers* change relative to *GenericPlayers*.

The ComputedAppraisals model was able to capture some of the bias in human observers’ emotion predictions for *SpecificPlayers*. Across all *SpecificPlayers*, the fit between the predicted difference $\Delta \hat{e}$ of the ComputedAppraisals model and the empirical difference Δe was: ccc=0.439 [0.424,0.449], Pearson r=0.471 [0.460,0.483]. However, human observers disagreed amongst themselves about how emotion predictions should be personalized for each *SpecificPlayer*. The emotions predicted for some players reliably differ from the emotions predicted for the *GenericPlayers*, whereas other players did not elicit reliable differences (Δe). We therefore separated the correlations in emotion prediction bias for each *SpecificPlayer* in figure 6*d*. Correlations are scaled by the total variation (see the electronic supplementary methods §S1l(ii)). The ComputedAppraisals model was better able to capture the relative difference in predicted emotions for the *SpecificPlayers* that evoked more reliably different emotion predictions. We hypothesize that the ComputedAppraisals model is mimicking human observers’ adjustment of emotion predictions, based on computed appraisals with personalized values and expectations.

Neither the InversePlanningLesion model nor the SocialLesion model were able to generate personalized emotion predictions (figure 6*d*,*e*). Despite predicting the expected emotion intensities nearly as well as the ComputedAppraisals model (figure 6*c*), the InversePlanningLesion model largely failed to predict how personalizing information biased emotion predictions relative to the generic players: ccc=0.089 [0.069,0.098], Pearson r=0.107 [0.083,0.118]. The SocialLesion model yielded a similarly low correlation: ccc=0.116 [0.116,0.116], Pearson r=0.146 [0.146,0.146].

## 7. Discussion

This work computationally models how observers predict other’s emotions. We formalize emotion prediction as a Bayesian intuitive theory of emotion by building on modelling work from psychology, computer science and behavioural economics. Integrating these approaches in a cognitively structured model enables us to investigate how observers infer, reason over and predict, abstract representations of other’s mental contents. A contribution of this work is in illustrating how to modularly combine theory-based computational models in a causal Bayesian framework. The three Modules (figure 1) implement general psychological hypotheses but are tailored to the context of the ‘Split or Steal’ game. Each could be improved to better capture human cognition both within this domain and in general.

Module (1) instantiates a hypothesis about the causal generative structure of the mind: how observers infer the unobservable mental contents likely to have motivated someone’s observed behaviour. The module simulates how observers reason about what preferences and beliefs caused a player’s action by inverting a richly structured generative model of approximately rational decision making. The aim is to infer abstract Theory of Mind representations from the observable features of a situation (the players’ actions and the pot size) by modelling observers’ intuitive theory of players’ minds. We define objective representations of the situations, which are psychologically relevant but do not depend on mental state inferences. We operationalize this using the Fehr & Schmidt [97] parametrization of social inequity aversion (electronic supplementary material, figure S2), which reflects salient dimensions of events in the ‘Split or Steal’ games and is also generally applicable to a large domain of dyadic interactions. The forward planning model simulates how players plan and execute behaviours based on their beliefs about the world and other people, and preferences for the objective situation features and other people’s beliefs. The forward model builds up distributions over actions by sampling players with varying preferences and beliefs from empirically derived priors. Bayesian inversion of the forward planning model yields inference of the preferences and beliefs that jointly motivated a player’s observed behaviour.

Any instantiation of Module (1) expresses a hypothesis about which preferences and beliefs are potentially at stake in a situation. For instance, if we hypothesized that observers thought that players were only concerned with maximizing their monetary reward, we would not include preferences for social equity, and defecting would be the only rational choice for simulated players. Similarly, if we hypothesized that observers thought that players were not managing their reputations, we would use the AnonymousGame model, rather than a model that simulates players’ preferences for how they are perceived by others. However, observers expect players to cooperate and report that players have preferences for what inferences others make about their values. Thus, to capture the social cognition that we hypothesize observers employ to reason about why a player chose to cooperate or defect, we instantiate a Bayesian Theory of Mind (BToM) model with preferences and beliefs over recursive representations of the monetary and social features of the situation and other people. Our instantiation captures observers’ judgements of the latent mental contents that motivated a player’s action (figure 2).

Prior BToM models have focused on belief-desire inference (so-called ‘propositional attitudes’ [130]) via Bayesian inverse planning. BToM models have been applied extensively to infer motivations using POMDPs and reinforcement learning in domains where agents interact with simple environments [15,39,42,44,131,132]. Our present work demonstrates how the machinery of inverse planning can be extended to reason over a more general Bayesian Theory of Mind that includes appraisals and emotions. To our knowledge, this is the most richly structured inverse planning model to date, and the first use of inverse planning to predict emotional reactions to subsequent events.

Module (2) instantiates a hypothesis about the computational basis of predicted emotional reactions: how observers reason about someone’s appraisal of events. The module computes how a simulated player would evaluate a new world state (the outcome of the game) caused by an event outside of the player’s intentional control (the opponent’s action) given the player’s preferences and beliefs. The specific computations are inspired by the general cognitive principles of Appraisal Theory. We compute achieved utilities, prediction errors and counterfactuals over monetary, social and reputational representations, incorporating the simulated player’s beliefs about what was going to happen, what did and did not happen, and how the player and the opponent could have changed what happened (figure 3).

To build a computational model that generates emotion predictions from contextual information, we use situations with quantitatively well-defined features. This allows us to investigate what abstract representations and computations observers use to translate situation information into emotion predictions. Choosing a well-studied social context enables us to adapt behavioural economic models of behaviour to model people’s intuitive theory of actions and reactions. Scaling this approach to less constrained contexts will require more general methods of computing abstract psychologically relevant representations of situations [133]. While a Prisoner’s Dilemma can be expressed as the players’ actions, the pot size, the rules of the game (in the case of ‘Split or Steal’, a public one-shot weak PD), the cognitively relevant features of most real-world social interactions are harder to parse.

Large language models have recently made impressive strides in capturing patterns of social cognition [25,26,134–137], and various neural architectures have been able to capture some patterns of human Theory of Mind in gridworld tasks [138–140]. However, these models have yet to approach the logical and causal reasoning capacity that humans develop by childhood [139,141,142]. Compared to neural architecture with minimal upfront structure, highly structured inverse planning models evidence greater sophistication and generalization, even in Theory of Mind tasks simple enough for infants [42–44]. In our view, capturing the breadth and sophistication of social cognition will require probabilistic generative models of the human intuitive Theory of Mind [143]. Advances in probabilistic programming, program synthesis and neurosymbolic methods [144–148] suggest that the relevant abstractions can be learned by combining theory-based inductive constraints and conducive experimental domains [63,149–152].

Module (3) instantiates a hypothesis about the structure of emotion concepts: how observers transform latent appraisal representations into emotion predictions (an example is shown in figure 5). In the present work, we target retrospective emotions (emotions that occur in response to the outcome of the game) and did not include prospective emotions that concern uncertain future events (e.g. *hope*, *fear*; see the electronic supplementary methods §S1e). Emotion concepts (functions of appraisal variables) are learned by finding a transformation between the joint distribution over computed appraisals and the joint distribution over empirical emotion judgements. An advantage of this approach is that the computational structure of social cognition can be reverse-engineered directly from emotion judgements. Because the learned appraisal structure is interpretable, the results can be compared with other emotion taxonomies. While it did not need to be the case, the learned appraisal structure is unique for each emotion, suggesting that these 20 emotions are conceptually distinct (figure 4).

A downside of this approach is that the learned structure is limited by the event context. A context that does not induce reliable variance in the computed appraisals or in the empirical emotion judgements will lead to poor identifiability. For instance, observers tended to judge all events in the ‘Split or Steal’ game as surprising, leading the model to capture low overall variance of *surprise* (figure 6*b*). Nonetheless, the model learned appropriate appraisal structure for the reliable empirical patterns. Observers judged players to be the most surprised when the players cooperated and their opponents defected (CD, figure 3*d*). Since simulated players do not tend to cooperate when they expect their opponents to defect (figure 2), players show large disadvantageous inequity aversion prediction errors when they are exploited. Thus, the model learned that *surprise* is a function of ${PE}_{DIA}^{\mathrm{base}}$ (figure 4). Model comparison validates this: the InversePlanningLesion model, which does not represent *why* a player chose an action, cannot capture *surprise* judgements at all (figure 6*b*).

Our work makes strong commitments to inverse planning as the mechanism by which observers abstract Theory of Mind representations from situations, and to computed appraisals as the cognitive basis of emotion understanding. There are many alternative ways to model the relationship between situations and emotion predictions. It would be possible to learn direct relationships between emotion judgements and situation features, like monetary reward and the players’ decisions [153], but this would describe emotions in terms of the choices players made and rewards they received without explaining why these features are psychologically relevant. To study how emotion judgements relate to abstract representations of situations, it would be possible to model emotion judgements based on empirical ratings of players’ appraisals, but this would not address how observers generate the appraisal ratings from contextual information [54,68]. Rather than relying on observers to generate appraisals from context, it would be possible to define appraisals based directly on the event context without using inverse planning, but this would not address how emotion predictions depend on a player’s intentions and motivations [4]. Finally, it would be possible to align a model’s appraisal computations with variables prescribed by Appraisal Theories in order to predict emotions based on the emotion concepts defined in terms of those appraisal variables [24]. However, this would limit the model to emotions that have been previously defined, and require one to hand code functions that transform Theory of Mind representations into prescribed appraisal variables, such as motivational relevance, goal congruence, controllability, probability and novelty [75].

Our goal is to reverse-engineer the human intuitive theory of emotion. In the present work, we focus on prediction, following the stance that forward causal reasoning is a core cognitive capacity that enables explanation and planning [154,155]. By illustrating how inverse planning over a Bayesian Theory of Mind can be extended to support a greater range of social cognitive reasoning, our work outlines a path towards building formal models of everyday social cognition. Our model of emotion prediction can be integrated with Bayesian models of expression interpretation [65], to capture abductive inference (causal reasoning about what events someone previously experienced) [3] and contextualized emotion attribution (integrating contextual and perceptual information to infer someone’s emotions) [4,156]. Emotion prediction is also a critical input to planning social interactions. People deliberately plan and choose actions in order to cause specific emotional reactions in their partners (with variable success). For a model of human emotional intelligence to capture commonsense reasoning about others’ past, present and future experiences, and human social interactions in general, the model must have the capacity to predict others’ emotional reactions to hypothetical events [8,13,14,46,113,157]. In characterizing the sophisticated latent reasoning involved in predicting emotions, our work suggests that even seemingly simple acts of emotion understanding involve abstract social reasoning.

## 8. Methods

This section describes how the ComputedAppraisals model learns a transformation from computed appraisals to emotion predictions. The extended Methods are given in the electronic supplementary materials, §S1.

### (a) Learning the latent appraisal structure of emotion concepts

We learn a sparse transformation from the joint distribution over computed appraisal variables to the joint distribution over emotion predictions. A transformation is described by a weights matrix β, intercepts vector b and covariance matrix Σ. The likelihood is given by,

8.1 $$L(\beta ,b,\Sigma ;E)=\prod_{e\in E}\text{P}(e|\beta ,b,\Sigma ),$$

where the matrix E is the set empirical emotion predictions for the *GenericPlayers*. When predicting a player’s emotions, observers were given the decisions of both players in the game and the pot size, such that each emotion vector e in this set is associated with a tuple of independent variables that defines the outcome of the game: $\left\langle a_{1},a_{2},pot\right\rangle$.

The PublicGame model generates a joint distribution over computed appraisals given the outcome of a game: $\text{P}(\psi |a_{1},a_{2},pot)$. The computed appraisal set Ψ comprises the ψ vectors sampled from the joint posterior for every combination of independent variables. We learn a transformation from computed appraisals (Ψ) to emotions (E) by maximizing the probability of observing the empirical emotion predictions under a uniformly weighted multivariate Gaussian mixture. For an empirical emotion prediction vector, e, which is associated with a given outcome, $\left\langle a_{1},a_{2},pot\right\rangle$:

8.2 $$\begin{array}{rl} \text{P}(e|\beta ,b,\Sigma ) & \;=\underset{\text{P}(\psi |a_{1},a_{2},pot)}{\text{E}}[P(e|\psi ,\beta ,b,\Sigma )]\\ & \;=\underset{\text{P}(\psi |a_{1},a_{2},pot)}{\text{E}}[N(e;\mu ={\mathrm{logit}}^{-1}(k\;\cdot \;\psi \;\cdot \;\beta +b),\Sigma )]\\ & \;\approx \frac{1}{N}\sum_{i}^{N}N(e;\mu ={\mathrm{logit}}^{-1}(k\;\cdot \;\psi_{i}\;\cdot \;\beta +b),\sigma^{2}\text{I}). \end{array}$$

We apply a logistic transformation to accommodate the [0,1] bounds of the empirical emotion predictions, with the steepness constant k set to 0.4. The Gaussian mixture is composed of N samples (indexed by i) from the posterior distribution of computed appraisals for a given game outcome. The Gaussian kernel uses a diagonal covariance matrix, formed from the variance vector $\sigma^{2}$ and the identity matrix I. Note that while this enforces orthogonal covariance between emotions in the kernel, the joint distribution over appraisals induces covariance between emotions in the final mixture. Thus, we fit all 20 emotions jointly, and the β weights are constrained by the covariance between simulated appraisals and by the empirical covariance between emotions.

### (b) Regularization and cross-validation

We induce a sparse solution by placing a Laplace prior on the β weights:

8.3 $${\left(\beta ,b,\Sigma \right)}^{\text{MAP}}=\underset{\beta ,b,\Sigma }{\mathrm{argmax}}\text{P}(E|\beta ,b,\Sigma )\;\text{P}{\left(\beta \right)}^{M},$$

where P(E∣β,b,Σ) is the likelihood from equation (8.1). The prior P(β) is given by β∼Laplace(0,τ), where τ is the scale hyperparameter, and adjusted by the number of empirical observations (E is composed of M emotion vectors) so that the importance of the data relative to the prior is constant for empirical datasets of different sizes. We estimate the Maximum *a posteriori* (MAP) parameters via gradient descent with $\ell_{1}$ regularization on every β weight. This contrasts with a common approach of seeking a sparse number of total predictors. Rather, each β weight is regularized independently, which allows different emotion concepts to be defined by different sets of computed appraisal variables. To improve feature selection and the interpretability of β weights, each computed appraisal variable is scaled to have unit standard deviation prior to model fitting.

To fit the scale of the Laplace prior (τ), we performed gridsearch and K-fold cross-validation on subsets of the *SpecificPlayers*. The emotion predictions used for analyses were generated for *SpecificPlayers* that were held out of the fitting process. The optimization procedure is detailed in the electronic supplementary methods §S1k, and described in brief here. For every considered value of the hyperparameter, we generated a posterior distribution over computed appraisals using the GenericPrior (described in the electronic supplementary methods §S1j) and fit the transformation parameters to the empirical predictions of the *GenericPlayers’* emotions. We then generated computed appraisal distributions for 15 *SpecificPlayers* using the SpecificPriors (described in the electronic supplementary methods §S1j(i)) and transformed these into emotion predictions using the transformation parameters that were fit to the *GenericPlayers*. The Laplace scale that provided the best generalization to the 15 *SpecificPlayers* in the cross-validation set was used to predict emotions for the five *SpecificPlayers* that were held out for testing. This was iterated to generate emotion predictions for all of the *SpecificPlayers*.

A learned transformation represents a likely point estimate of the posterior distribution over the parameters. We ran the optimization many times using random initializations to estimate expectations and confidence intervals of the β parameter weights.

## Data Availability

All behavioural data, models and analyses are available at: https://github.com/daeh/computed-appraisals. The data are provided in electronic supplementary material [158].

## Footnotes

1 This payoff structure is similar to a one-shot Prisoner’s Dilemma (PD): the payoffs are symmetrical for the players, the players make their decisions without knowledge of the other player’s choice, and it is never in either player’s financial interest to cooperate. However, because both being defected on (CD) and mutual defection (DD) confer the same payoff ($0), this game has been referred to as a ‘weak’ PD [2].

2 Indeed, Appraisal Theory was initially proposed [95] and later developed [96] to explain how the same event might evoke different emotions, in different people, on different occasions.

3 The probability weighting term is based on the softmax distribution in equation (3.3). Since the simulated player has now observed which choice the opponent made, the player’s belief about the opponent’s action is updated to reflect the player’s knowledge: $\pi_{a_{2}}\to a_{2}$. This gives a measure of how likely the player would have made the other choice ($\neg a_{1}$), if the player had known which action the opposing player was going to choose.

4 Whereas Pearson’s r is insensitive to whether samples differ in intercept or scale, Lin’s ccc penalizes the model’s deviations from human judgements by the mean squared error. Also see the electronic supplementary methods §S1l(i).

## References

[1] van den Assem MJ, van Dolder D, Thaler RH. 2012 Split or steal? Cooperative behavior when the stakes are large. Manag. Sci. **58**, 2-20. (10.1287/mnsc.1110.1413)

[2] Rapoport A. 1988 Experiments with N-person social traps I: prisoner’s dilemma, weak prisoner’s dilemma, volunteer’s dilemma, and largest number. J. Conflict Resolut. **32**, 457-472. (10.1177/0022002788032003003)

[3] Houlihan SD, Ong D, Cusimano M, Saxe R. 2022 Reasoning about the antecedents of emotions: Bayesian causal inference over an intuitive theory of mind. In *Proc. of the 44th Annual Conf. of the Cognitive Science Society, Toronto, Canada, 27–30 July*, vol. 44, pp. 854–861.

[4] Ong DC, Zaki J, Goodman ND. 2015 Affective cognition: exploring lay theories of emotion. Cognition **143**, 141-162. (10.1016/j.cognition.2015.06.010) 26160501

[5] Chen Z, Whitney D. 2019 Tracking the affective state of unseen persons. Proc. Natl Acad. Sci. USA **116**, 7559-7564. (10.1073/pnas.1812250116) 30814221 PMC6462097

[6] Tenenbaum JB, Griffiths TL, Kemp C. 2006 Theory-based Bayesian models of inductive learning and reasoning. Trends Cogn. Sci. **10**, 309-318. (10.1016/j.tics.2006.05.009) 16797219

[7] Tenenbaum JB, Kemp C, Griffiths TL, Goodman ND. 2011 How to grow a mind: statistics, structure, and abstraction. Science **331**, 1279-1285. (10.1126/science.1192788) 21393536

[8] Saxe R, Houlihan SD. 2017 Formalizing emotion concepts within a Bayesian model of theory of mind. Curr. Opin. Psychol. **17**, 15-21. (10.1016/j.copsyc.2017.04.019) 28950962 PMC5637274

[9] Weiner B. 1986 An attributional theory of motivation and emotion. New York, NY: Springer. 3903815

[10] Hareli S. 2014 Making sense of the social world and influencing it by using a naïve attribution theory of emotions. Emot. Rev. **6**, 336-343. (10.1177/1754073914534501)

[11] de Melo CM, Carnevale PJ, Read SJ, Gratch J. 2014 Reading people’s minds from emotion expressions in interdependent decision making. J. Pers. Soc. Psychol. **106**, 73-88. (10.1037/a0034251) 24079297

[12] Yongsatianchot N, Marsella S. 2016 Integrating model-based prediction and facial expressions in the perception of emotion. In *Artificial General Intelligence* (eds B Steunebrink, P Wang, B Goertzel), pp. 234–243. Cham, Switzerland: Springer International Publishing.

[13] Ong DC, Zaki J, Goodman ND. 2019 Computational models of emotion inference in theory of mind: a review and roadmap. Top. Cogn. Sci. **11**, 338-357. (10.1111/tops.12371) 30066475 PMC7077035

[14] Wu Y, Schulz LE, Frank MC, Gweon H. 2021 Emotion as information in early social learning. Curr. Dir. Psychol. Sci. **30**, 468-475. (10.1177/09637214211040779)

[15] Baker CL, Jara-Ettinger J, Saxe R, Tenenbaum JB. 2017 Rational quantitative attribution of beliefs, desires and percepts in human mentalizing. Nat. Hum. Behav. **1**, 598. (10.1038/s41562-017-0064)

[16] Baker CL, Saxe R, Tenenbaum JB. 2009 Action understanding as inverse planning. Cognition **113**, 329-349. (10.1016/j.cognition.2009.07.005) 19729154

[17] Jara-Ettinger J, Gweon H, Schulz LE, Tenenbaum JB. 2016 The naïve utility calculus: computational principles underlying commonsense psychology. Trends Cogn. Sci. **20**,589-604. (10.1016/j.tics.2016.05.011) 27388875

[18] Marsella S, Gratch J, Petta P. 2010 Computational models of emotion. In *A blueprint for affective computing-a sourcebook and manual* (eds KR Scherer, T Bänziger, E Roesch), pp. 21–46. Oxford, UK: Oxford University Press.

[19] Mellers BA, Schwartz A, Ho K, Ritov I. 1997 Decision affect theory: emotional reactions to the outcomes of risky options. Psychol. Sci. **8**, 423-429. (10.1111/j.1467-9280.1997.tb00455.x)

[20] Battigalli P, Dufwenberg M, Smith A. 2015 Frustration and Anger in games. SSRN scholarly Paper ID 2591839 Social Science Research Network Rochester, NY. (10.2139/ssrn.2591839)

[21] Elliott CD. 1992 The affective reasoner: a process model of emotions in a multiagent system. PhD thesis, Northwestern University.

[22] Ortony A, Clore GL, Collins A. 1990 The cognitive structure of emotions. Cambridge,UK: Cambridge University Press.

[23] Si M, Marsella SC, Pynadath DV. 2010 Modeling appraisal in theory of mind reasoning. Auton. Agents Multi Agent Syst. **20**, 14-31. (10.1007/s10458-009-9093-x)

[24] Alfonso B, Pynadath DV, Lhommet M, Marsella S. 2015 Emotional perception for updating agents’ beliefs. In *2015 Int. Conf. on Affective Computing and Intelligent Interaction (ACII), Xi'an, China*, pp. 201–207. IEEE.

[25] Rashkin H, Sap M, Allaway E, Smith NA, Choi Y. 2018 Event2mind: commonsense inference on events, intents, and reactions. In *Proc. of the 56th Annu. Meeting of the Association for Computational Linguistics (Volume 1: Long Papers)*, pp. 463–473. Melbourne, Australia: Association for Computational Linguistics.

[26] Sap M, Rashkin H, Chen D, LeBras R, Choi Y. 2019 Social IQa: commonsense reasoning about social interactions. In *EMNLP, Hong Kong, China,* pp. 4463–4473.

[27] Gerstenberg T, Tenenbaum JB. 2017 Intuitive theories. In *Oxford handbook of causal reasoning* (ed. M Waldmannn), pp. 515–548. Oxford, UK: Oxford University Press.

[28] Carey S. 2009 The origin of concepts. Oxford, UK: Oxford University Press.

[29] Gopnik A, Wellman HM. 2012 Reconstructing constructivism: causal models, Bayesian learning mechanisms, and the theory theory. Psychol. Bull. **138**, 1085-1108. (10.1037/a0028044) 22582739 PMC3422420

[30] Murphy GL, Medin DL. 1985 The role of theories in conceptual coherence. Psychol. Rev. **92**, 289-316. (10.1037/0033-295X.92.3.289) 4023146

[31] Zaki J, Bolger N, Ochsner K. 2009 Unpacking the informational bases of empathic accuracy. Emotion **9**, 478-487. (10.1037/a0016551) 19653768 PMC6558959

[32] Thornton MA, Tamir DI. 2017 Mental models accurately predict emotion transitions. Proc. Natl Acad. Sci. USA **114**, 5982-5987. (10.1073/pnas.1616056114) 28533373 PMC5468631

[33] Zhao Z, Thornton MA, Tamir DI. 2022 Accurate emotion prediction in dyads and groups and its potential social benefits. Emotion **22**, 1030-1043. (10.1037/emo0000890) 32940486 PMC8064741

[34] Gilbert DT, Pinel EC, Wilson TD, Blumberg SJ, Wheatley TP. 1998 Immune neglect: a source of durability bias in affective forecasting. J. Pers. Soc. Psychol. **75**, 617-638. (10.1037/0022-3514.75.3.617) 9781405

[35] Pollmann MMH, Finkenauer C. 2009 Empathic forecasting: how do we predict other people’s feelings? Cogn. Emot. **23**, 978-1001. (10.1080/02699930802264895)

[36] Kleiman-Weiner M, Shaw A, Tenenbaum JB. 2017 Constructing social preferences from anticipated judgements: when impartial inequity is fair and why? In *Proc. of the 39th Annu. Conf. of the Cognitive Science Society, London, UK, 26–29 July*, vol. 39, pp. 676–681.

[37] Kleiman-Weiner M, Gerstenberg T, Levine S, Tenenbaum JB. 2015 Inference of intention and permissibility in moral decision making. In *Proc. of the 37th Annu. Conf. of the Cognitive Science Society, Pasadena, CA, 22–25 July*, vol. 37, pp. 1123–1128.

[38] Jern A, Lucas CG, Kemp C. 2017 People learn other people’s preferences through inverse decision-making. Cognition **168**, 46-64. (10.1016/j.cognition.2017.06.017) 28662485 PMC5572562

[39] Jara-Ettinger J, Schulz LE, Tenenbaum JB. 2020 The naïve utility calculus as a unified, quantitative framework for action understanding. Cogn. Psychol. **123**, 101334. (10.1016/j.cogpsych.2020.101334) 32738590

[40] Evans O, Stuhlmüller A, Goodman ND. 2016 Learning the preferences of ignorant, inconsistent agents. In *30th AAAI Conf. on Artificial Intelligence, AAAI 2016* pp. 323–329. Oxford, UK: University of Oxford.

[41] Kryven M, Ullman T, Cowan W, Tenenbaum JB. 2016 Outcome or strategy? A Bayesian model of intelligence attribution. In *Proc. of the 38th Annu. Conf. of the Cognitive Science Society, Philadelphia, PA, 10–13 August*, vol. 38. 10.1111/cogs.13041 34490914

[42] Zhi-Xuan T, Gothoskar N, Pollok F, Gutfreund D, Tenenbaum JB, Mansinghka VK. 2022 Solving the baby intuitions benchmark with a hierarchically Bayesian theory of mind. (10.48550/ARXIV.2208.02914)

[43] Shu T, Bhandwaldar A, Gan C, Smith K, Liu S, Gutfreund D, Spelke E, Tenenbaum J, Ullman T. 2021 Agent: a benchmark for core psychological reasoning. In *Proc. of the 38th Int. Conf. on Machine Learning, vol. 139. Proceedings of Machine Learning Research* (eds M Meila, T Zhang), pp. 9614–9625. PMLR.

[44] Shum M, Kleiman-Weiner M, Littman ML, Tenenbaum JB. 2019 Theory of minds: understanding behavior in groups through inverse planning. Proc. AAAI Conf. Artif. Intell. **33**, 6163-6170. (10.1609/aaai.v33i01.33016163)

[45] Albrecht SV, Stone P. 2018 Autonomous agents modelling other agents: a comprehensive survey and open problems. Artif. Intell. **258**, 66-95. (10.1016/j.artint.2018.01.002)

[46] Gurney N, Marsella S, Ustun V, Pynadath DV. 2022 Operationalizing theories of theory of mind: a survey. In *Computational theory of mind for human-machine teams* (eds N Gurney,G Sukthankar), pp. 3–20. Cham, Switzerland: Springer Nature.

[47] De Bruyn A, Bolton GE. 2008 Estimating the influence of fairness on bargaining behavior. Manag. Sci. **54**, 1774-1791. (10.1287/mnsc.1080.0887)

[48] Dufwenberg M, Gneezy U. 2000 Measuring beliefs in an experimental lost wallet game. Games Econ. Behav. **30**, 163-182. (10.1006/game.1999.0715)

[49] Frijda NH. 1986 The emotions. New York, NY: Cambridge University Press.

[50] Lazarus RS. 1991 Emotion and adaptation. New York, NY: Oxford University Press.

[51] Moors A. 2014 Flavors of appraisal theories of emotion. Emot. Rev. **6**, 303-307. (10.1177/1754073914534477)

[52] Moors A, Ellsworth PC, Scherer KR, Frijda NH. 2013 Appraisal theories of emotion: state of the art and future development. Emot. Rev. **5**, 119-124. (10.1177/1754073912468165)

[53] Scherer KR. 2005 Appraisal theory. In *Handbook of cognition and emotion *(eds T Dalgleish, MJ Power), pp. 637–663. Chichester, UK: John Wiley & Sons, Ltd.

[54] Skerry AE, Saxe R. 2015 Neural representations of emotion are organized around abstract event features. Curr. Biol. **25**, 1945-1954. (10.1016/j.cub.2015.06.009) 26212878 PMC4824044

[55] Wu Y, Baker CL, Tenenbaum JB, Schulz LE. 2018 Rational inference of beliefs and desires from emotional expressions. Cogn. Sci. **42**, 850-884. (10.1111/cogs.12548) 28986938 PMC6033160

[56] Weiner B. 1987 The social psychology of emotion: applications of a naive psychology. J. Soc. Clin. Psychol. **5**, 405-419. (10.1521/jscp.1987.5.4.405)

[57] Siemer M, Reisenzein R. 2007 The process of emotion inference. Emotion **7**, 1-20. (10.1037/1528-3542.7.1.1) 17352558

[58] Wondra JD, Ellsworth PC. 2015 An appraisal theory of empathy and other vicarious emotional experiences. Psychol. Rev. **122**, 411-428. (10.1037/a0039252) 25961468

[59] Gratch J, de Melo CM. 2019 Inferring intentions from emotion expressions in social decision making. In *The social nature of emotion expression : what emotions can tell us about the world* (eds U Hess, S Hareli), pp. 141–160. Cham, Switzerland: Springer International Publishing.

[60] Hareli S, Hess U. 2010 What emotional reactions can tell us about the nature of others: an appraisal perspective on person perception. Cogn. Emot. **24**, 128-140. (10.1080/02699930802613828)

[61] Scherer KR, Mortillaro M, Rotondi I, Sergi I, Trznadel S. 2018 Appraisal-driven facial actions as building blocks for emotion inference. J. Pers. Soc. Psychol. **114**, 358-379. (10.1037/pspa0000107) 29461080

[62] de Melo CM, Terada K. 2020 The interplay of emotion expressions and strategy in promoting cooperation in the iterated prisoner’s dilemma. Sci. Rep. **10**, 14959. (10.1038/s41598-020-71919-6) 32917943 PMC7486426

[63] Ong DC, Soh H, Zaki J, Goodman ND. 2021 Applying probabilistic programming to affective computing. IEEE Trans. Affect. Comput. **12**, 306-317. (10.1109/TAFFC.2019.2905211) 34055236 PMC8162129

[64] Teo DWH, Ang ZY, Ong DC. 2022 Modeling causal inference from emotional displays. In *Proc. of the 44th Annu. Conf. of the Cognitive Science Society, Toronto, Canada, 27–30 July*, vol. 44, pp. 2200–2206.

[65] Houlihan SD. 2022 A computational framework for emotion understanding. PhD thesis, Massachusetts Institute of Technology Cambridge, MA, USA.

[66] Houlihan SD, Kleiman-Weiner M, Tenenbaum JB, Saxe R. 2018 A generative model of people’s intuitive theory of emotions: inverse planning in rich social games. In *Proc. of the 40th Annu. Conf. of the Cognitive Science Society, Madison, WI, 25–28 July*, vol. 40.

[67] Houlihan SD, Kleiman-Weiner M, Tenenbaum JB, Saxe R. 2018 A generative model of people’s intuitive theory of emotions: inverse planning in rich social games. In *Cognitive computational neuroscience, Philadelphia, USA*.

[68] Scherer KR, Meuleman B. 2013 Human emotion experiences can be predicted on theoretical grounds: evidence from verbal labeling. PLoS ONE **8**, e58166-8. (10.1371/journal.pone.0058166) 23483988 PMC3590138

[69] Battigalli P, Dufwenberg M. 2007 Guilt in games. Am. Econ. Rev. **97**, 170-176. (10.1257/aer.97.2.170)

[70] van Baar JM, Chang LJ, Sanfey AG. 2019 The computational and neural substrates of moral strategies in social decision-making. Nat. Commun. **10**, 1-14. (10.1038/s41467-019-09161-6) 30940815 PMC6445121

[71] Sell A *et al.* 2017 The grammar of anger: mapping the computational architecture of a recalibrational emotion. Cognition **168**, 110-128. (10.1016/j.cognition.2017.06.002) 28668649

[72] Sznycer D. 2019 Forms and functions of the self-conscious emotions. Trends Cogn. Sci. **23**, 143-157. (10.1016/j.tics.2018.11.007) 30583948

[73] Sznycer D, Sell A, Lieberman D. 2021 Forms and functions of the social emotions. Curr. Dir. Psychol. Sci. **30**, 292-299. (10.1177/09637214211007451)

[74] Gratch J, Marsella SC. 2014 Appraisal models. In *The Oxford Handbook of Affective Computing*, pp. 54–67. USA: Oxford University Press.

[75] Smith CA, Lazarus RS. 1990 Emotion and adaptation. In *Handbook of personality: theory and research*, pp. 609–637. New York, NY: The Guilford Press.

[76] Roseman IJ. 2001 A model of appraisal in the emotion system: integrating theory, research, and applications. In *Appraisal processes in emotion: theory, methods, research* (eds KR Scherer, A Schorr, T Johnstone), Series in Affective Science, pp. 68–91. New York, NY: Oxford University Press.

[77] Clore GL, Ortony A. 2013 Psychological construction in the OCC model of emotion. Emot. Rev. **5**, 335-343. (10.1177/1754073913489751) 25431620 PMC4243519

[78] Cowen AS, Keltner D. 2020 What the face displays: mapping 28 emotions conveyed by naturalistic expression. Am. Psychol. **75**, 349-364. (10.1037/amp0000488) 31204816 PMC6917997

[79] Yu Z, Zhang C. 2015 Image based static facial expression recognition with multiple deep network learning. In *Proc. of the 2015 ACM on Int. Conf. on Multimodal Interaction ICMI ’15*, pp. 435–442. New York, NY: Association for Computing Machinery. (10.1145/2818346.2830595)

[80] Abdul-Mageed M, Ungar L. 2017 EmoNet: fine-grained emotion detection with gated recurrent neural networks. In *Proc. of the 55th Annu. Meeting of the Association for Computational Linguistics (Volume 1: Long Papers)*, pp. 718–728. Vancouver, Canada: Association for Computational Linguistics. (10.18653/v1/P17-1067)

[81] Li S, Deng W. 2020 Deep facial expression recognition: a survey. IEEE Trans. Affect. Comput. **13**, 1195-1215. (10.1109/TAFFC.2020.2981446)

[82] Feng K, Chaspari T. 2020 A review of generalizable transfer learning in automatic emotion recognition. Front. Comput. Sci. **2**, 9. (10.3389/fcomp.2020.00009)

[83] Lin C, Bulls LS, Tepfer L, Vyas AD, Thornton MA. 2023 Advancing naturalistic affective science with deep learning. (10.31234/osf.io/j5q9h) PMC10514024 37744976

[84] Mittal T, Guhan P, Bhattacharya U, Chandra R, Bera A, Manocha D. 2020 EmotiCon: context-aware multimodal emotion recognition using Frege’s principle. In *2020 IEEE/CVF Conf. on Computer Vision and Pattern Recognition (CVPR)*, pp. 14 222–14 231. Seattle, WA: IEEE. (10.1109/CVPR42600.2020.01424)

[85] Mittal T, Mathur P, Bera A, Manocha D. 2021 Affect2MM: affective analysis of multimedia content using emotion causality. In *Proc. of the IEEE/CVF Conf. on Computer Vision and Pattern Recognition (CVPR), 19–25 June*, pp. 5661–5671.

[86] Parry G, Vuong Q. 2021 Deep affect: using objects, scenes and facial expressions in a deep neural network to predict arousal and valence values of images. Preprint PsyArXiv. (10.31234/osf.io/t9p3f)

[87] Wei Z, Zhang J, Lin Z, Lee JY, Balasubramanian N, Hoai M, Samaras D. 2020 Learning visual emotion representations from web data. In *2020 IEEE/CVF Conf. on Computer Vision and Pattern Recognition (CVPR)*, pp. 13103–13112. Seattle, WA, USA: IEEE. (10.1109/CVPR42600.2020.01312)

[88] Kosti R, Alvarez JM, Recasens A, Lapedriza A. 2017 Emotion recognition in context. In *2017 IEEE Conf. on Computer Vision and Pattern Recognition (CVPR), Honolulu, HI, 21–26 July*, pp. 1960–1968. IEEE. (10.1109/cvpr.2017.212) 31095475

[89] Falk A, Fehr E, Fischbacher U. 2003 On the nature of fair behavior. Econ. Inq. **41**, 20-26. (10.1093/ei/41.1.20)

[90] Kiyonari T, Tanida S, Yamagishi T. 2000 Social exchange and reciprocity: confusion or a heuristic? Evol. Hum. Behav. **21**, 411-427. (10.1016/S1090-5138(00)00055-6) 11146306

[91] Hayashi N, Ostrom E, Walker J, Yamagishi T. 1999 Reciprocity, trust, and the sense of control: a cross-societal study. Ration. Soc. **11**, 27-46. (10.1177/104346399011001002)

[92] de Melo CM, Marsella S, Gratch J. 2016 People do not feel guilty about exploiting machines. ACM Trans. Comput. Hum. Interact. **23**, 1-17. (10.1145/2890495)

[93] Zeelenberg M, van Dijk WW, van der Pligt J, Manstead AS, van Empelen P, Reinderman D. 1998 Emotional reactions to the outcomes of decisions: the role of counterfactual thought in the experience of regret and disappointment. Organ. Behav. Hum. Decis. Process. **75**, 117-141. (10.1006/obhd.1998.2784) 9719660

[94] Mandel D. 2003 Counterfactuals, emotions, and context. Cogn. Emot. **17**, 139-159. (10.1080/02699930302275) 29715740

[95] Arnold MB. 1960 Emotion and personality. New York, NY: Columbia University Press.

[96] Lazarus RS. 1966 Psychological stress and the coping process. New York, NY: McGraw-Hill.

[97] Fehr E, Schmidt KM. 1999 A theory of fairness, competition, and cooperation. Q. J. Econ. **114**, 817-868. (10.1162/003355399556151)

[98] Bolton GE, Ockenfels A. 2000 ERC: a theory of equity, reciprocity, and competition. Am. Econ. Rev. **90**, 166-193. (10.1257/aer.90.1.166)

[99] Blake PR *et al.* 2015 The ontogeny of fairness in seven societies. Nature **528**, 258-261. (10.1038/nature15703) 26580018

[100] Henrich J *et al.* 2005 ‘Economic Man’ in cross-cultural perspective: behavioral experiments in 15 small-scale societies. Behav. Brain Sci. **28**, 795-815. (10.1017/S0140525X05000142) 16372952

[101] Kahneman D, Tversky A. 1979 Prospect theory: an analysis of decision under risk. Econometrica **47**, 263-291. (10.2307/1914185)

[102] Tversky A, Kahneman D. 1992 Advances in prospect theory: cumulative representation of uncertainty. J. Risk Uncertain. **5**, 297-323. (10.1007/BF00122574)

[103] Balaz V, Bačová V, Drobná E, Dudeková K, Adamík K. 2013 Testing prospect theory parameters. Ekon. Cas. **61**, 655-671.

[104] Luce RD. 1959 Individual choice behavior: a theoretical analysis. New York, NY: Wiley.

[105] Manstead ASR, Fischer AH. 2001 Social appraisal: the social world as object of and influence on appraisal processes. In *Appraisal processes in emotion: theory, methods, research *(eds KR Scherer, A Schorr, T Johnstone), Series in Affective Science, pp. 221–232. New York, NY: Oxford University Press.

[106] Sally D. 1995 Conversation and cooperation in social dilemmas: a meta-analysis of experiments from 1958 to 1992. Ration. Soc. **7**, 58-92. (10.1177/1043463195007001004)

[107] Rutledge RB, Skandali N, Dayan P, Dolan RJ. 2014 A computational and neural model of momentary subjective well-being. Proc. Natl Acad. Sci. USA **111**, 12 252-12 257. (10.1073/pnas.1407535111) PMC4143018 25092308

[108] Knutson B, Taylor J, Kaufman M, Peterson R, Glover G. 2005 Distributed neural representation of expected value. J. Neurosci. **25**, 4806. (10.1523/JNEUROSCI.0642-05.2005) 15888656 PMC6724773

[109] Joffily M, Coricelli G. 2013 Emotional valence and the free-energy principle. PLoS Comput. Biol. **9**, e1003094. (10.1371/journal.pcbi.1003094) 23785269 PMC3681730

[110] Eldar E, Niv Y. 2015 Interaction between emotional state and learning underlies mood instability. Nat. Commun. **6**, 6149. (10.1038/ncomms7149) 25608088 PMC5338993

[111] Emanuel A, Eldar E. 2023 Emotions as computations. Neurosci. Biobehav. Rev. **144**, 104977. (10.1016/j.neubiorev.2022.104977) 36435390 PMC9805532

[112] Seth AK. 2013 Interoceptive inference, emotion, and the embodied self. Trends Cogn. Sci. **17**, 565-573. (10.1016/j.tics.2013.09.007) 24126130

[113] Barrett LF. 2017 The theory of constructed emotion: an active inference account of interoception and categorization. Soc. Cogn. Affect. Neurosci. **12**, 1-23. (10.1093/scan/nsw154) 27798257 PMC5390700

[114] Wu Y, Gweon H. 2021 Preschool-aged children jointly consider others’ emotional expressions and prior knowledge to decide when to explore. Child Dev. **92**, 862-870. (10.1111/cdev.13585) 34033118

[115] Modirshanechi A, Brea J, Gerstner W. 2022 A taxonomy of surprise definitions. J. Math. Psychol. **110**, 102712. (10.1016/j.jmp.2022.102712)

[116] Mellers B, Schwartz A, Ritov I. 1999 Emotion-based choice. J. Exp. Psychol.: Gen. **128**, 332-345. (10.1037/0096-3445.128.3.332)

[117] Gilovich T, Medvec VH. 1995 The experience of regret: what, when, and why. Psychol. Rev. **102**, 379-395. (10.1037/0033-295X.102.2.379) 7740094

[118] Coricelli G, Rustichini A. 2010 Counterfactual thinking and emotions: regret and envy learning. Phil. Trans. R. Soc. B **365**, 241-247. (10.1098/rstb.2009.0159) 20026462 PMC2827450

[119] McMullen MN, Markman KD. 2002 Affective impact of close counterfactuals: implications of possible futures for possible pasts. J. Exp. Soc. Psychol. **38**, 64-70. (10.1006/jesp.2001.1482)

[120] Gleicher F, Kost KA, Baker SM, Strathman AJ, Richman SA, Sherman SJ. 1990 The role of counterfactual thinking in judgements of affect. Pers. Soc. Psychol. Bull. **16**, 284-295. (10.1177/0146167290162009)

[121] Gummerum M, Cribbett C, Nogueira Nicolau A, Uren R. 2013 Counterfactual reasoning and moral emotion attribution. Eur. J. Dev. Psychol. **10**, 144-158. (10.1080/17405629.2012.756394)

[122] Johnson JT. 1986 The knowledge of what might have been: affective and attributional consequences of near outcomes. Pers. Soc. Psychol. Bull. **12**, 51-62. (10.1177/0146167286121006)

[123] Asaba M, Ong DC, Gweon H. 2019 Integrating expectations and outcomes: preschoolers’ developing ability to reason about others’ emotions. Dev. Psychol. **55**, 1680-1693. (10.1037/dev0000749) 31094560

[124] Sweeny K, Vohs KD. 2012 On near misses and completed tasks: the nature of relief. Psychol. Sci. **23**, 464-468. (10.1177/0956797611434590) 22477104

[125] Niedenthal PM, Tangney JP, Gavanski I. 1994 “If only I weren’t” versus “If only I hadn’t”: distinguishing shame and guilt in counterfactual thinking. J. Pers. Soc. Psychol. **67**, 585-595. (10.1037/0022-3514.67.4.585) 7965606

[126] Roseman IJ. 2013 Appraisal in the emotion system: coherence in strategies for coping. Emot. Rev. **5**, 141-149. (10.1177/1754073912469591)

[127] Lin LIK. 1989 A concordance correlation coefficient to evaluate reproducibility. Biometrics **45**, 255-268. (10.2307/2532051) 2720055

[128] Jenkins AC, Karashchuk P, Zhu L, Hsu M. 2018 Predicting human behavior toward members of different social groups. Proc. Natl Acad. Sci. USA **115**, 9696-9701. (10.1073/pnas.1719452115) 30201708 PMC6166817

[129] Oosterhof NN, Todorov A. 2008 The functional basis of face evaluation. Proc. Natl Acad. Sci. USA **105**, 11 087-11 092. (10.1073/pnas.0805664105) 18685089 PMC2516255

[130] Dennett DC. 1998 The intentional stance. A bradford book, Cambridge, MA: MIT Press.7 printing edition.

[131] Jara-Ettinger J. 2019 Theory of mind as inverse reinforcement learning. Curr. Opin. Behav. Sci. **29**, 105-110. (10.1016/j.cobeha.2019.04.010)

[132] Wu SA, Wang RE, Evans JA, Tenenbaum JB, Parkes DC, Kleiman-Weiner M. 2021 Too many cooks: bayesian inference for coordinating multi-agent collaboration. Top. Cogn. Sci. **13**,414-432. (10.1111/tops.12525) 33829670

[133] Allen KR *et al.* 2023 Using games to understand the mind. (10.31234/osf.io/hbsvj)

[134] Suresh V, Ong DC. 2021 Using knowledge-embedded attention to augment pre-trained language models for fine-grained emotion recognition. In *2021 9th Int. Conf. on Affective Computing and Intelligent Interaction (ACII), Nara, Japan, 28 September*, pp. 1–8. IEEE.

[135] Yang K, Lee D, Whang T, Lee S, Lim H. 2019 EmotionX-KU: BERT-Max based contextual emotion classifier. (http://arxiv.org/abs/1906.11565)

[136] Hwang JD, Bhagavatula C, Le Bras R, Da J, Sakaguchi K, Bosselut A, Choi Y. 2021 (Comet-) atomic 2020: on symbolic and neural commonsense knowledge graphs. Proc. AAAI Conf. Artif. Intell. **35**, 6384-6392. (10.1609/aaai.v35i7.16792)

[137] Kosinski M. 2023 Theory of mind may have spontaneously emerged in large language models. (http://arxiv.org/abs/2302.02083)

[138] Rabinowitz N, Perbet F, Song F, Zhang C, Eslami SMA, Botvinick M. 2018 Machine theory of mind. In *Proc. of the 35th Int. Conf. on Machine Learning, vol. 80, Proceedings of Machine Learning Research, Stockholm, Sweden, 10–15 July* (eds J Dy, A Krause), pp. 4218–4227. PMLR.

[139] Gandhi K, Stojnic G, Lake BM, Dillon MR. 2021 Baby intuitions benchmark (bib): discerning the goals, preferences, and actions of others. In *Advances in neural information processing systems*, vol. 34 (eds M Ranzato, A Beygelzimer, Y Dauphin, P Liang, JW Vaughan), pp. 9963–9976. Red Hook, NY: Curran Associates, Inc. 10.1016/j.cognition.2023.105406 36801603

[140] Nguyen D, Nguyen P, Le H, Do K, Venkatesh S, Tran T. 2023 Memory-augmented theory of mind network. (http://arxiv.org/abs/2301.06926)

[141] Sap M, LeBras R, Fried D, Choi Y. 2023 Neural theory-of-mind? On the limits of social intelligence in large LMs. (http://arxiv.org/abs/2210.13312)

[142] Ullman T. 2023 Large language models fail on trivial alterations to theory-of-mind tasks. (http://arxiv.org/abs/2302.08399)

[143] Houlihan SD, Tenenbaum JB, Saxe R. 2021 Linking models of theory of mind and measures of human brain activity. In *The neural basis of mentalizing* (eds M Gilead, KN Ochsner), pp. 209–235. Cham, Switzerland: Springer International Publishing.

[144] Lake BM, Salakhutdinov R, Tenenbaum JB. 2015 Human-level concept learning through probabilistic program induction. Science **350**, 1332-1338. (10.1126/science.aab3050) 26659050

[145] Ellis K, Wong C, Nye M, Sable-Meyer M, Cary L, Morales L, Hewitt L, Solar-Lezama A, Tenenbaum JB. 2020 Dreamcoder: growing generalizable, interpretable knowledge with wake-sleep Bayesian program learning. (http://arxiv.org/abs/2006.08381) 10.1098/rsta.2022.0050 37271169

[146] Ellis K, Albright A, Solar-Lezama A, Tenenbaum JB, O’Donnell TJ. 2022 Synthesizing theories of human language with Bayesian program induction. Nat. Commun. **13**, 5024. (10.1038/s41467-022-32012-w) 36042196 PMC9427767

[147] Le TA, Collins KM, Hewitt L, Ellis K, Tenenbaum JB. 2022 Hybrid memoised wake-sleep: approximate inference at the discrete-continuous interface. In *Int. Conf. on Learning Representations, 25–29 April*.

[148] Feinman R, Lake BM. 2021 Learning task-general representations with generative neuro-symbolic modeling. In *9th Int. Conf. on Learning Representations, ICLR 2021, Virtual Event, Austria, 3–7 May 2021*.

[149] Lake BM, Ullman TD, Tenenbaum JB, Gershman SJ. 2017 Building machines that learn and think like people. Behav. Brain Sci. **40**, e253. (10.1017/S0140525X16001837) 27881212

[150] Rule JS, Tenenbaum JB, Piantadosi ST. 2020 The child as hacker. Trends Cogn. Sci. **24**, 900-915. (10.1016/j.tics.2020.07.005) 33012688 PMC7673661

[151] Kleiman-Weiner M, Sosa F, Thompson B, van Opheusden B, Griffiths TL, Gershman S, Cushman F. 2020 Downloading culture.zip: social learning by program induction. In *Proc. of the 42nd Annu. Conf. of the Cognitive Science Society, 29 July–1 August*, vol. 42, pp. 1667–1673.

[152] Tsividis PA, Loula J, Burga J, Foss N, Campero A, Pouncy T, Gershman SJ, Tenenbaum JB. 2021 Human-level reinforcement learning through theory-based modeling, exploration, and planning. (http://arxiv.org/abs/2107.12544) 10.1098/rsta.2024.0529 42130435

[153] Levine EE, Barasch A, Rand D, Berman JZ, Small DA. 2018 Signaling emotion and reason in cooperation. J. Exp. Psychol.: Gen. **147**, 702-719. (10.1037/xge0000399) 29745712

[154] Pearl J. 2009 Causal inference in statistics: an overview. Stat. Surv. **3**, 96-146. (10.1214/09-SS057)

[155] Gerstenberg T, Goodman ND, Lagnado DA, Tenenbaum JB. 2021 A counterfactual simulation model of causal judgements for physical events. Psychol. Rev. **128**, 936-975. (10.1037/rev0000281) 34096754

[156] Anzellotti S, Houlihan SD, Liburd Jr. S, Saxe R. 2021 Leveraging facial expressions and contextual information to investigate opaque representations of emotions. Emotion **21**, 96-107. (10.1037/emo0000685) 31580092

[157] Ho MK, Saxe R, Cushman F. 2022 Planning with theory of mind. Trends Cogn. Sci. **26**, 959-971. (10.1016/j.tics.2022.08.003) 36089494

[158] Houlihan SD, Kleiman-Weiner M, Hewitt LB, Tenenbaum JB, Saxe R. 2023 Emotion prediction as computation over a generative theory of mind. Figshare. (10.6084/m9.figshare.c.6631138) PMC10239682 37271174

[159] Goodman ND, Stuhlmüller A. 2014 The design and implementation of probabilistic programming languages. (http://dippl.org)

